# Rollable Single‐Piece Thermoelectric Generators at Cryogenic Temperature Fabricated with High‐performance CNT Films Achieved by Doping Modulation

**DOI:** 10.1002/advs.202515688

**Published:** 2025-10-21

**Authors:** Zihan Zhu, Kuncai Li, Xin Hao, Xu Dai, Jing Wang, Fenfen Yin, Hong Wang

**Affiliations:** ^1^ Frontier Institute of Science and Technology Xi'an Jiaotong University Xi'an 710054 China; ^2^ State Key Laboratory of Multiphase Flow in Power Engineering Xi'an Jiaotong University Xi'an 710054 China; ^3^ School of Energy and Power Engineering Xi'an Jiaotong University Xi'an 710054 China; ^4^ Shaanxi Canon Flexible Thermoelectric Technology Co., Ltd Xi'an Shaanxi Province 710000 China

**Keywords:** cryogenic temperature, high power factor, miniature GPS locator, mini‐thermoelectric power station, single‐piece TEG

## Abstract

Thermoelectric generators (TEGs) can convert heat to electricity through all‐solid‐state structure, which makes them suitable for energy recovery in cold environments. However, cryogenic TEG development faces dual challenges: severe power factor (PF) degradation and interfacial failures due to thermal expansion mismatch at module‐electrode junctions. In this work, a high PF is reported up to 10.5 mW m^−1^ K^−2^ at room temperature in carbon nanotube (CNT) films achieved by a developed material‐processing strategy with an annealing treatment method to carefully optimize the chlorosulfonic acid (CSA) doping level included. This PF value is close to some of state‐of‐the‐art inorganic thermoelectric materials at room temperature, which varies in 20% over a temperature range of 100–298 K that is superior to most of the state‐of‐the‐art inorganic thermoelectric materials (25%–70%). Spectra demonstrate that the CSA doping is a physical adsorption process, which is successfully fitted by the pseudo‐second‐order kinetic model. In addition, a single‐piece rollable TEG is fabricated to mitigate interfacial stresses, and this TEG maintained exceptional flexibility and structural integrity under cryogenic thermal cycling. Furthermore, a miniature thermoelectric power station is fabricated through integrating the rolled TEGs, which demonstrates the promising application of CNT based TEGs in cold environments.

## Graphical abstract

High power factor CNT of 10.5 mW m^−1^ K^−2^ at 298 K achieved by a developed material‐processing strategy exhibited a small variation by 20% in the temperature range of 100–298 K which surpassed most inorganic materials (25‐70%). The CNT film‐based single‐piece TEG showed exceptional ultra‐low temperature tolerance and great flexibility, which is promising for energy recovery in cold environments.

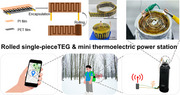

## 1 Introduction

Thermoelectric generators (TEGs) have shown significant potential at waste heat recovery due to their ability to directly convert heat into electricity via an all‐solid‐state structure.^[^ ^1^, ^2^, ^3^, ^4^, ^5^ ^]^ TEGs operate independently of light conditions, unlike photovoltaic devices that rely on solar irradiation,^[^ ^6^, ^7^, ^8^ ^]^ making them uniquely suited for extreme environments, such as Arctic/Antarctic polar nights, deep space exploration,^[^ ^9^, ^10^, ^11^, ^12^ ^]^ and industrial applications like liquid natural gas (LNG) cold energy recovery.^[^ ^13^, ^14^, ^15^ ^]^ Their lack of moving parts and liquid fluids further enhances reliability, reduces maintenance needs, and enables deployment in remote or harsh settings.

However, current low‐temperature TEGs (<300 K) primarily rely on rigid, heavy inorganic materials.^[^ ^16^, ^17^, ^18^ ^]^ The widespread adoption of these TEGs in cryogenic conditions (typically below 200K) faces two critical challenges. First, the thermoelectric (TE) performance of conventional materials deteriorates sharply at low temperatures. The power factor (*PF = S^2^σ*, *S* is the Seebeck coefficient, *σ* is the electrical conductivity) of state‐of‐the‐art thermoelectric materials often degrades markedly^[^ ^18^, ^19^, ^20^ ^]^ due to suppressed charge carrier mobility and increased carrier freeze‐out effects,^[^ ^21^, ^22^ ^]^ which greatly reduces power output density^[^ ^17^ ^]^ and renders TEGs inefficient for cryogenic applications. For instance, CsBi_4_Te_6_^[^ ^23^ ^]^ Nb_4_SiTe_4_^[^ ^24^ ^]^ and SnSe^[^ ^25^ ^]^ drops by ≈25%, ≈40% and ≈70% when cooled from room temperature to 100 K, respectively. Second, interfacial reliability issues arise from thermal expansion mismatch between dissimilar materials at module‐electrode junctions, leading to mechanical delamination and accelerated contact resistance growth, which ultimately compromise long‐term device performance and structure integrity. When inorganic thermoelectric devices operate at low temperature range, the deterioration of the solder layer can lead to device failure, which reduces the maximum output power of the device.^[^ ^26^ ^]^ Therefore, this gap underscores the critical need to develop thermoelectric materials that synergistically combine high power factors with robust cryogenic durability and structural stability. Such materials would not only enhance power output density but also enable reliable operation of TEGs in environments ranging from 100 to 400 K, a primary prerequisite for applications in Arctic/Antarctic polar nights, deep space exploration, and LNG cold energy recovery.

Carbon nanotube (CNT) has a low coefficient of thermal expansion in the range of 10^−6^/K,^[^ ^27^ ^]^ one tenth of that for traditional inorganic thermoelectric materials, making them ideal for thermoelectric energy harvesting at extreme cold environments. Theoretically, an isolated CNT has high power factor (PF ≈100 mW m^−1^ K^−2^) enabled by quantum confinement effects that narrow carrier distribution in individual CNTs.^[^ ^28^, ^29^, ^30^, ^31^ ^]^ Progress has been achieved in the past years in terms of the Seebeck coefficient of metallic CNTs, which increases from <10 µV K^−1^ to ≈60µV K^−1^ by shifting the Fermi level to approach the 1D van Hove singularity (vHs) of metallic CNTs via tuning the doping level.^[^ ^32^, ^33^, ^34^, ^35^ ^]^ Recently, CNT films and fibers have exhibited PF values approaching theoretical power factor of an isolated CNT, such as 1–9.3 mWm^−1^K^−2^ for films^[^ ^34^, ^35^, ^36^, ^37^ ^]^ and 14.5 mWm^−1^K^−2^ for fibers.^[^ ^38^ ^]^ Additionally, CNTs are light weight and flexible, which enable the CNT based TEGs to conform to irregular surface geometries, thereby minimizing thermal contact resistance at heat source interfaces.^[^ ^39^ ^]^ Meanwhile, innovations like the single‐piece TEG design with p‐n modules on one piece of CNT film has further enhanced mechanical robustness and reduced internal resistance compared to traditional π‐structured TEGs.^[^ ^35^ ^]^ Despite these advances, research on CNT film‐based TEGs at cryogenic temperatures is scarce.

In this work, we reported a high PF up to 10.5 mW m^−1^ K^−2^ at ≈298 K in CNT films achieved by a developed material‐processing strategy with an annealing treatment method to carefully optimize the chlorosulfonic acid (CSA) doping level. This PF value is close to some of the state‐of‐the‐art inorganic thermoelectric materials at room temperature,^[^ ^25^, ^40^ ^]^ which varies by 20% in a temperature range of 100–298 K which is superior to most of the state‐of‐the‐art inorganic thermoelectric materials (25%–70%).^[^ ^23^, ^24^, ^25^ ^]^ Raman spectroscopy and X‐ray photoelectron spectroscopy (XPS) confirmed that the CSA doping is a physical adsorption process, which is successfully fitted by the pseudo‐second‐order kinetic model. To demonstrate practical applicability, a compact rollable single‐piece TEG was fabricated with 6 p‐n module pairs by selectively patterning p‐type CSA doped CNT films with n‐type dopant polyethyleneimine (PEI). The obtained single‐piece TEG was encapsulated using low thermally conductive substrate materials: polyethylene terephthalate (PET) films and Polyimide (PI) films. Notably, the rolled TEG matched the output power of its planar counterpart across both near‐room‐temperature and cryogenic environments. Furthermore, we designed a mini thermoelectric power station by integrating the rolled TEG with tunable internal resistance (via series‐parallel circuity) and thermal storage tanks. This system generated volt‐scale electrical outputs of 0.42 V (at ∆T = 60 K)‐1.4 V (at ∆T = 220 K) and delivered high output powers in 14 µW (at ∆T = 60 K)‐175 µW (at ∆T = 220 K) range using either hot water (≈86 °C) or liquid nitrogen (−196 °C) as thermal storage media. Stable operation for over 2.5 h enabled sufficient energy production to power small electronics, including an electronic timer, an LED light and a miniature GPS locator. This work demonstrates a promising application of CNT based TEGs in cold environments.

## 2 Results and Discussion

### 2.1 Thermoelectric Material and Device Preparation Process

**Figure 1:**
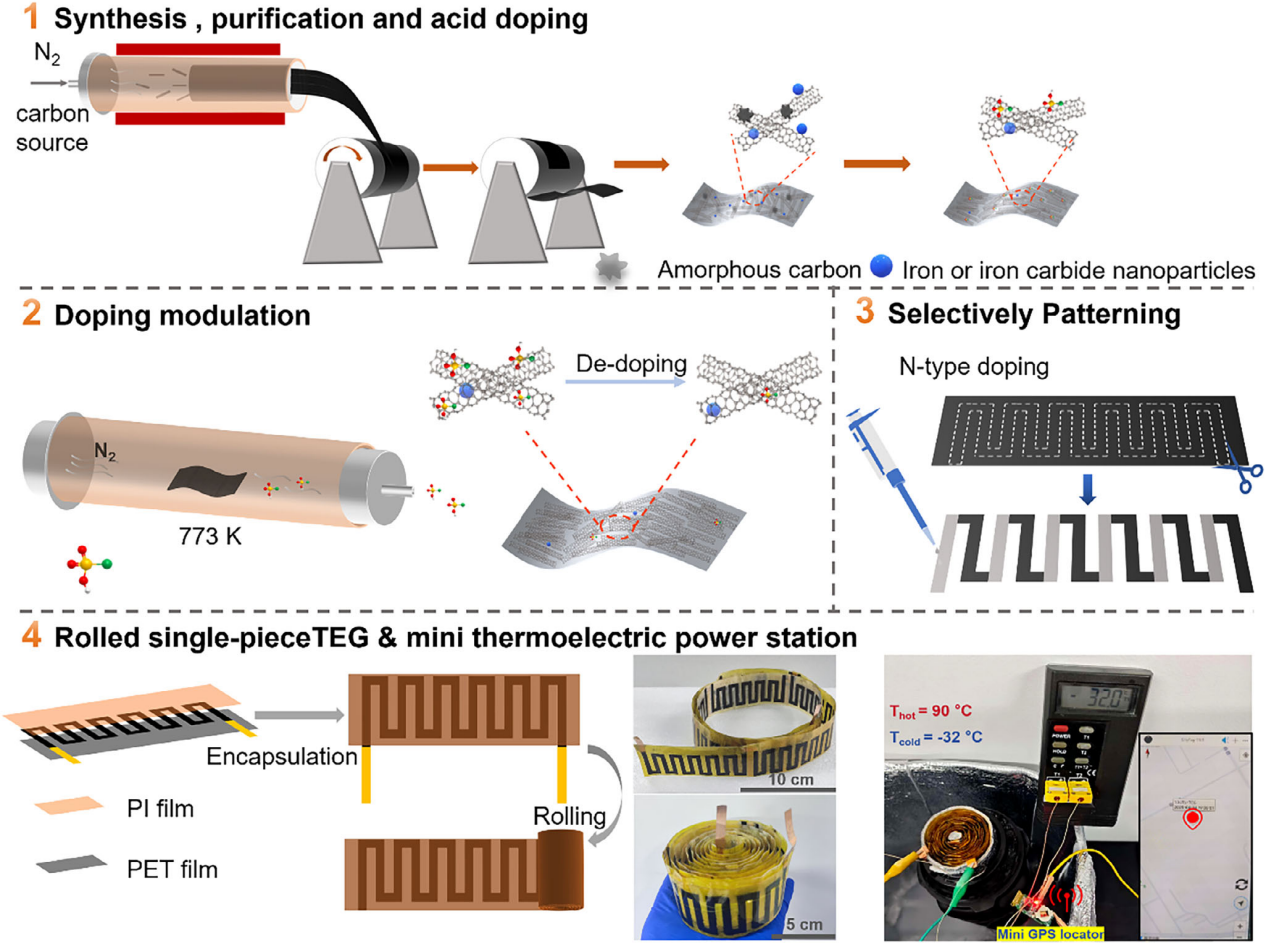
Thermoelectric materials and device preparation process. Illustration showing the preparation of high‐performance CNT films and the fabrication of the mini thermoelectric power station.

**Figure** **1** outlines the developed CNT film preparation process, the fabrication of rolled single‐piece TEGs and an integrated mini thermoelectric power station. Highly conductive macroscopic CNT films were synthesized via a floating catalysts chemical vapor deposition (FCCVD) method according to the literature,^[^ ^34^, ^35^, ^36^ ^]^ followed by purification and doping with chlorosulfonic acid (CSA) (see Supporting information for experimental details).

Transmission electron microscopy revealed that the films comprised a mixture of double‐walled and multi‐walled CNTs (Figure S1, Supporting Information). XPS analysis confirmed the presence of Fe and Fe/c nanoparticles encapsulated within the CNTs (Figure S2, Supporting Information), consistent with previous works.^[^ ^35^, ^36^ ^]^ To enhance the PF of CNT films, CSA‐doped CNT films were thermally annealed at 773 K under N_2_ atmosphere to achieved controlled de‐doping. After that, a rollable single‐piece TEG was prepared by selectively patterning the high PF p‐type CNT films, which was then encapsulated by low thermally conductive substrate PET films and PI films. Finally, a mini thermoelectric power station was designed by integrating the rolled single‐piece TEG and a heat/cold storage tank for long‐term usage in practical applications. The generated voltage and power by this mini thermoelectric power station were demonstrated to be sufficient to support small electronics.

### 2.2 CSA Doping Level Optimizing for High TE Performance p‐Type CNT Films

**Figure 2:**
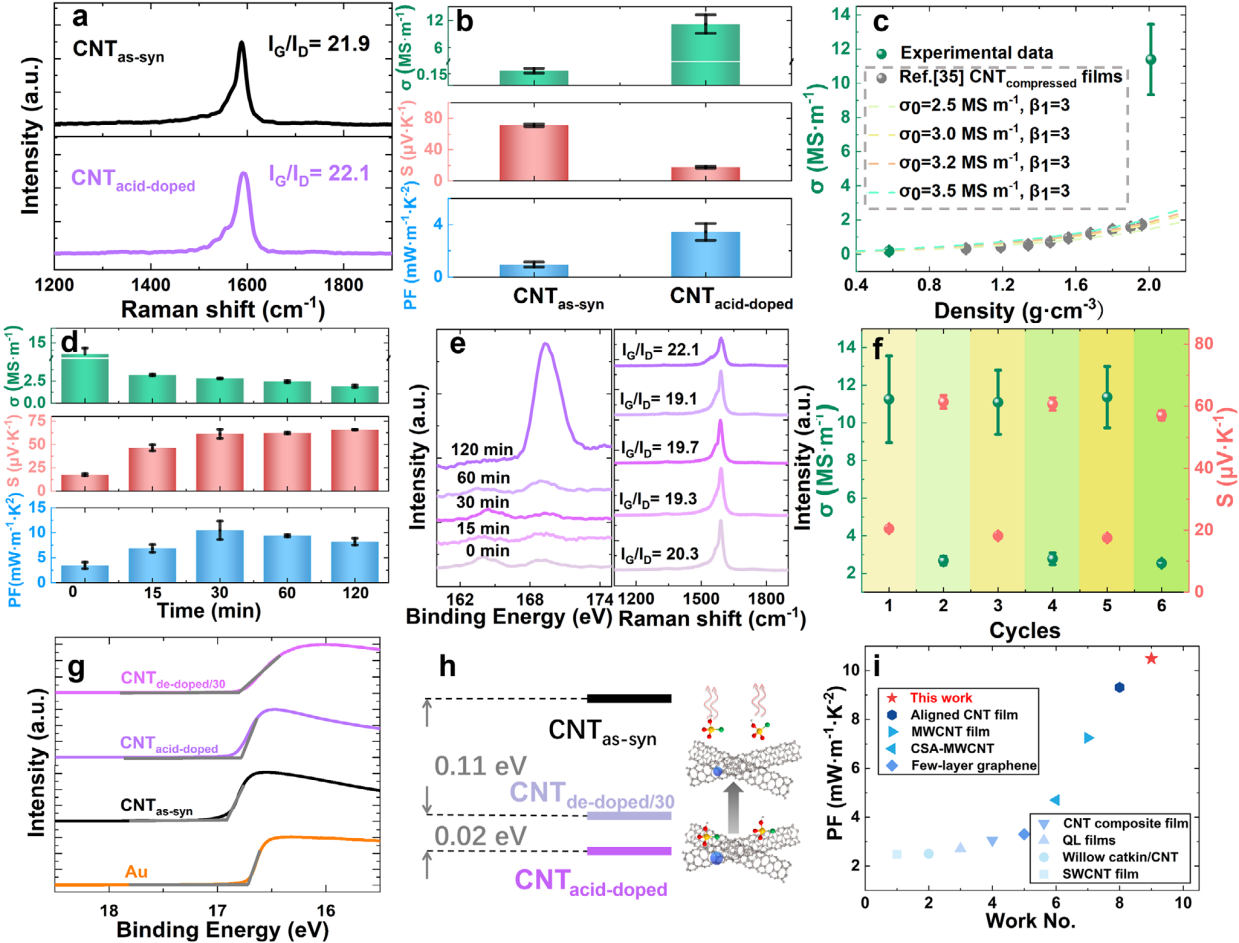
CSA doping level optimizing for high TE performance p‐type CNT films. a) Raman spectra of the CNT_as‐syn_ films and the CNT_acid‐doped_ films. b) The *σ*, *S* and PF of the CNT_as‐syn_ films and the CNT_acid‐doped_ films at ≈298 K. c) Density‐dependent electrical conductivity with Maxwell‐Eucken theoretical prediction (dashed line) d) The σ, S and PF of the CNT_acid‐doped_ films varied with the de‐doping time at ≈298 K. e) The Raman spectra and S 2p peak in XPS spectra of the CNT_acid‐doped_ films varied with the de‐doping time. f) Cyclic stability of σ and S through three doping‐dedoping iterations g) UPS results of Au, the CNT_as‐syn_ films, the CNT_acid‐doped_ films and the CNT_de‐doped/30_ films. h) The relative work function changes of the CNT_as‐syn_ films, the CNT_acid‐doped_ films and the CNT_de‐doped/30_ films which were obtained from Figure 2g. i) Comparison of the maximum PF of the CNT_de‐doped/30_ films at room temperature with that of p‐type non‐inorganic involved flexible films in literature.

The thermoelectric properties analysis of CNT films during the material preparation process were discussed in **Figure** **2**. Raman spectra showed that both the as‐synthesized CNT (CNT_as‐syn_) films and the acid doped CNT (CNT_acid‐doped_) had a good quality with high G‐band to D‐band intensity ratios (I_G_: I_D_) of 21.9 and 21.2 (Figure 2a), respectively. The films displayed anisotropic alignment along the rolling direction (Figure 1), conformed by polarized Raman measurements showing G‐band intensity ratios (I_G//_: I_G⊥_) of 2.7 (CNT_as‐syn_) and 2.8 (CNT_acid‐doped_) as shown in Figure S3 (Supporting Information). Please note that the electrical conductivity mentioned in the following sections referred to the electrical conductivity in the parallel direction unless specified. All the film thicknesses were identified by cross‐sectional scanning electron microscopy (SEM) images as shown in Figures S4 and S5(Supporting Information).

Figure 2b showed the electrical properties evolution of CNT films upon acid doping. Acid doping increased the electrical conductivity from 0.19±0.03 to 11.2±2.05 MS m^−1^ while reducing the Seebeck coefficient from 71 to 17 µV K^−1^. The significant enhancement in the electrical conductivity yielded a higher PF of CNT_acid‐doped_ than that in CNT_as‐syn_. To further understand significant enhancement in electrical conductivity of CNT_acid‐doped_, density analysis via Maxwell‐Eucken's equation^[^ ^35^ ^]^ linked electrical conductivity to film compaction. Theoretically, the electrical conductivity was proportional to the density of CNT films as shown in the Maxwell‐Eucken's equation:

(1) $$\sigma_{D}=\sigma_{0}\;\times \frac{D}{D_{0}+\beta_{1}\left(D_{0}-D\right)}$$

*D* was the density of a porous CNT film, and σ_*D*_ was the electrical conductivity of CNT films with a density of *D*, σ_0_ was the electrical conductivity of CNT films packed ideally in theory, *D_0_* was the density of CNT films packed ideally in theory, and β_1_ was the constant number determined by the conditions of the pores. The value of β_1_ was between 1.0 and 3.0 when the shape of the pores was almost spherical style.^[^ ^35^, ^41^, ^42^ ^]^

Figure 2c showed the variation of the electrical conductivity with the density for the CNT films prepared via a cold‐compressing method, which fitted well with the Equation (1). The CNT_acid‐doped_ had a higher density of 2.01 g cm^−3^ than that of 0.58 g cm^−3^ for CNT_as‐syn_. However, the electrical conductivity of CNT_acid‐doped_ far exceeded that of CNT_as‐syn_, which was off the theoretical lines, confirming that physically dense packing was not the main reason in enhancing the electrical conductivity of CNT_acid‐doped_. As the CNT quality was nearly un‐changed, confirmed by the I_G_: I_D_ of CNT films upon acid doping, and the CNT_compressed_ films^[^ ^35^ ^]^ had a similar CNT alignment in the films with the I_G//_:I_G⊥_ of 2.3^[^ ^37^ ^]^ close to that for the CNT_acid‐doped_ films (I_G//_:I_G⊥_ = 2.8), it was reasonable to conclude that acid doping had a dominate role in enhancing the electrical conductivity of CNT_acid‐doped_.

To optimize the PF values, CNT_acid‐doped_ films were then annealed at 773 K under N_2_ atmosphere. The annealing temperature was screened as shown in Figure S6 (Supporting Information). The trade‐off relationship tween electrical conductivity and Seebeck coefficient was shown in Figure 2d. Electrical conductivity of the CNT_acid‐doped_ films decreased from 11.2 to 3.2 MS m^−1^ after the CNT_acid‐doped_ films were annealed for 15 min, which then reached a platform at ≈1.9 MS m^−1^ until 120 min. While the Seebeck coefficient increased after the CNT_acid‐doped_ films were annealed, which reached a value of 62 µV K^−1^ at the annealing time of 60 min and then increased a little to 65 µV K^−1^ at the annealing time of 120 min (Figure 2d). The maximum PF of 10.5 mW m^−1^ K^−2^ at ∼298 K was achieved when the CNT_acid‐doped_ films were annealed at 773 K for 30 min (CNT_de‐doped/30_). SEM images indicated that the CNT_de‐doped/30_ films became porous with holes in the surfaces (Figure S7, Supporting Information) as compared to these non‐heated CNT_as‐syn_ films (Figure S4, Supporting Information). The films thickness of CNT_de‐doped/30_ films also slightly increased from ≈250 nm to ≈350 nm, which resulted in the decrease of the density of 2.01 g cm^−3^ for the CNT_acid‐doped_ films to 1.16 g cm^−3^ for the CNT_de‐doped/30_ films. The electrical conductivity of the CNT_de‐doped/30_ films was ≈2.8 MS m^−1^, which was still ≈6.5 times higher than the CNT_compressed_ films with the same density as shown in Figure 2c (σ(CNT_de‐doped/30_ films): σ(CNT_compressed_ films) = 6.5). It revealed that the CSA residues still played some role in improving the electrical conductivity of the CNT_de‐doped/30_ films. The electric conductivity and the Seebeck coefficient of CNT_de‐doped/30_ films were double‐checked and the optical image taken from a computer screen was shown in Figure S8 (Supporting Information).

Raman and XPS results confirmed that the doping of CNT films was reversible via CSA's physical adsorption. As shown in Figure 2e, annealing CNT_acid‐doped_ films at 773 K for 15 min (CNT_de‐doped/15_) resulted in slight decrease in the I_G_: I_D_ ratio while maintaining a high value of 19.1, demonstrating effective preservation of CNT structural integrity during de‐doping. Prolonged annealing time up to 120 min caused minimal variation in the I_G_: I_D_ ratio (19.1‐22.1), strongly suggesting that CSA predominantly interacts with CNTs through physical adsorption rather than chemical bonding.

XPS analysis in Figure 2e revealed a progressive decrease in sulfur content (S 2p peak at 169 eV) within CNT_acid‐doped_ films during thermal annealing process at 773 K, correlating with the electrical conductivity reduction shown in Figures S9a (Supporting Information). This correlation indicated that S concentration served as a qualitative indicator of doping‐level variation, given CSA's role as the primary dopant. Although both HCl and CSA were used in processing, thorough post‐HCl washing effectively removed chloride species, evidenced by the weak Cl 2p signal at 198.5 eV that disappeared after CSA treatment (Figure S10, Supporting Information). These observations confirmed CSA's dominance in the doping process. The proportional relationship between S 2p concentration and electrical conductivity enabled successful fitting of CSA desorption kinetics using a pseudo‐second‐order model.^[^ ^43^, ^44^ ^]^

(2) $$\sigma =\left(k_{PSO}\sigma_{0}^{2}+\sigma_{0}t\right)/t$$

where *k_PSO_* is the pseudo‐second‐order rate constant, σ_0_ was the electrical conductivity of CNT_acid‐doped_ films after de‐doping. *t* was the annealing time. Excellent agreement between experimental data and calculated fitting curves (Figure S9b, Supporting Information) confirmed the physical adsorption mechanism.

Remarkably, CSA re‐doping restored both electrical conductivity and Seebeck coefficient to original levels, with consistent recovery observed over three cycles (Figure 2f). This reversible behavior further validated CSA's physical adsorption dominance. The observed Seebeck‐electrical conductivity correlation (Figure S11, Supporting Information) aligned with the Kang‐Snyder model for organic thermoelectrics, demonstrating doping characteristics analogous to polymeric thermoelectric materials.

UV photoelectron spectroscopy (UPS) was performed to show the work function evolution during CNT_acid‐doped_ film de‐doping (Figure 2g). CNT_as‐syn_ films exhibited a work function lower than pure Au's. Acid doping induced a 0.11 eV downward shift from the vacuum level (Figure 2h), confirming p‐type doping that correlated with enhanced electrical conductivity and reduced Seebeck coefficient (Figure 2b). Annealing CNT_acid‐doped_ film at 773 K for 30 min (CNT_de‐doped/30_) partially reversed this shift (0.02 eV recovery), corresponding to electrical conductivity decrease and Seebeck coefficient increase (Figure 2d).

Hall measurements proved inaccurate for determining carrier mobility and concentration in CNT films due to quantum confinement effects^[^ ^45^, ^46^ ^]^ and interference from residual magnetic Fe nanoparticles (Figures S2 and S12, Supporting Information). Thermogravimetric analysis confirmed persistent Fe residues in both pristine CNT_as‐syn_ and doped CNT_acid‐doped_ films. To elucidate electrical conductivity evolution during de‐doping, we calculated weighted mobility following established methodology.^[^ ^37^ ^]^ This analysis identified carrier concentration reduction as the primary driver of electrical conductivity changes, with detailed mechanistic interpretation provided in Supporting Information.

The de‐doped CNT_de‐doped/30_ films achieved a high PF of 10.5 mW m^−1^ K^−2^ at ∼298K among CNT based‐thermoelectric films (Figure 2i), surpassing state‐of‐the‐art p‐type flexible organic thermoelectric materials while rivaling inorganic thermoelectric materials like BiSbTe (≈4.1 mW m^−1^ K^−2^),^[^ ^47^ ^]^ PbSe (≈4.2 mW m^−1^ K^−2^),^[^ ^48^ ^]^ etc. Comprehensive comparisons were tabulated in Table S1 (Supporting Information). Notably, these de‐doped CNT_de‐doped/30_ films also maintained exceptional air stability, retaining 94% electrical conductivity and 96% Seebeck coefficient after 60‐day ambient exposure (Figure S13, Supporting Information), demonstrating both high performance and environmental durability unmatched in organic thermoelectric systems.

### 2.3 Temperature‐Dependent TE Performance and Cryogenic Temperature Tolerance of CNT_de‐Doped/30_ Films

**Figure 3:**
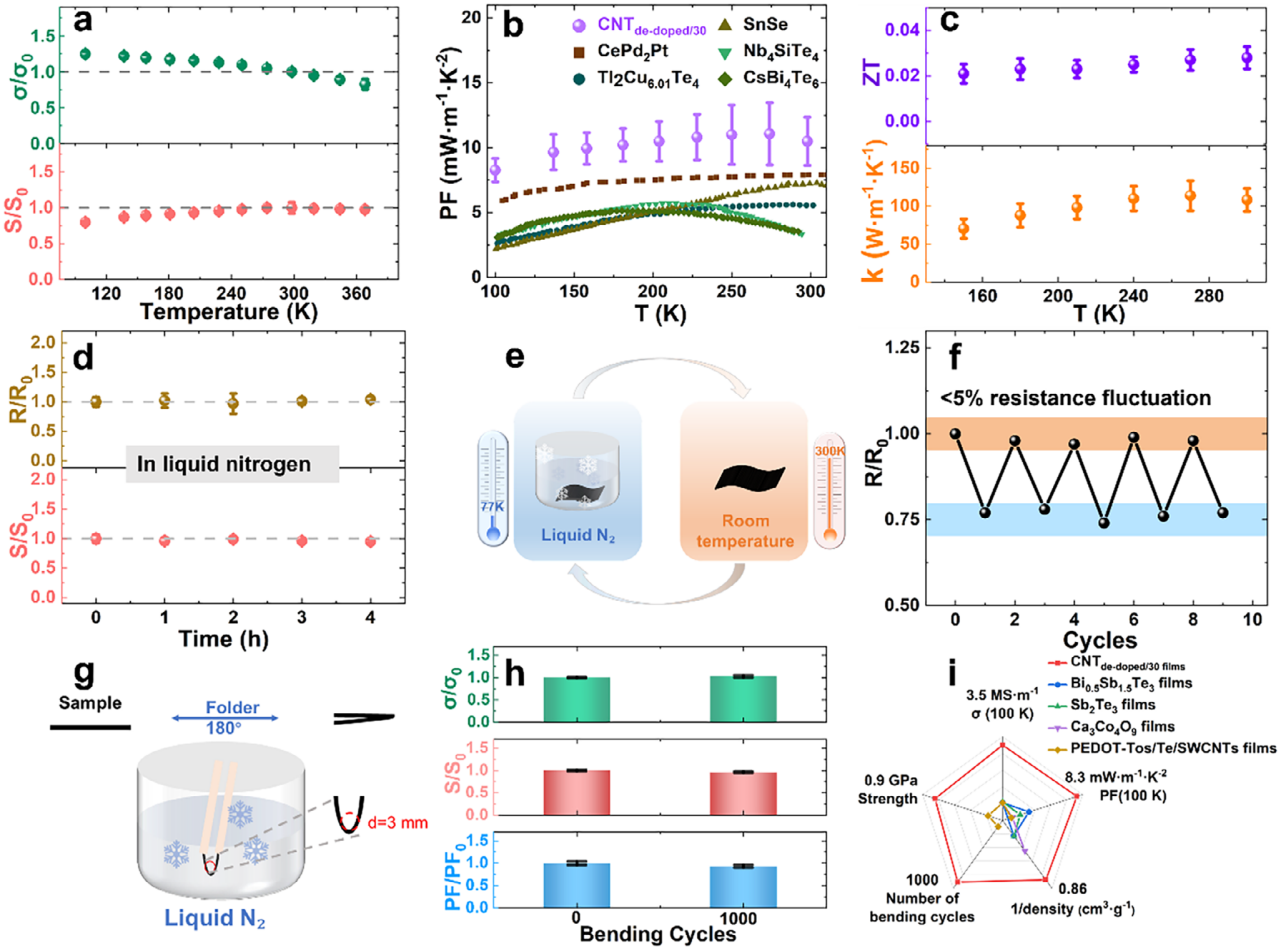
Temperature‐dependent TE performance and cryogenic temperature tolerance of CNT_de‐doped/30_ films a) Temperature‐dependent of σ and S of the CNT_de‐doped/30_ films. b) Comparison of the PF of the CNT_de‐doped/30_ films with that of p‐type inorganic thermoelectric materials at low temperature in literature.^[^ ^23^, ^24^, ^25^, ^40^, ^57^ ^]^ c) Temperature‐dependence of thermal conductivity and ZT of the CNT_de – doped/30_ films. d) The resistance and Seebeck coefficient of the CNT_de‐doped/30_ films as a function of soaking time in liquid nitrogen. e) The illustration and f) Variations in resistance of the CNT_de‐doped/30_ when switching the environmental temperature between 77 and 300 K. g) The illustration of the CNT_de‐doped/30_ films bending in liquid nitrogen and h) Variations in σ, S and PF of the CNT_de‐doped/30_ films after bending in liquid nitrogen. i) Comparison of comprehensive performance of the CNT _de – doped/30_ films with p‐type thermoelectric films at ultra‐low temperature in literature.

The CNT_de‐doped/30_ films exhibited unique cryogenic thermoelectric behavior (100–368 K) as shown in **Figure** **3**a. Electrical conductivity displayed metal‐like temperature dependence, increasing proportional with thermal elevation and fitting well to established electron‐phonon scattering model (Figure S14, Supporting Information), a characteristic previously observed in acid‐doped CNT fibers.^[^ ^49^ ^]^ Conversely, Seebeck coefficient exhibited inverse temperature dependence, decreasing by ≈20% from 298 to 100 K. Notably a high p‐type power factor of 8.3 mW m^−1^ K^−2^ persisted at 100 K, demonstrating exceptional low‐temperature performance retention despite electrical conductivity/Seebeck coefficient trade‐offs. Figure 3b demonstrated the cryogenic thermoelectric power factor stability of CNT_de‐doped/30_ films, maintaining high PF across an ultra‐wide temperature range (100–298 K). Notably, these films outperformed state‐of‐the‐art inorganic thermoelectric films in the 100–298 K range, achieving PF values 1.3–3.8 times higher than benchmark SnSe and CsBi_4_Te_6_ systems, which varies by 20% in a temperature range of 100–298 K which is superior to most of the state‐of‐the‐art inorganic thermoelectric materials (25%–70%).^[^ ^23^, ^24^, ^25^ ^]^ To fully evaluate the cryogenic thermoelectric performance of CNT_de‐doped/30_ films, the temperature dependent of thermal conductivity was tested in the range of 150–298 K as shown in Figure 3c. Thermoelectric analysis of CNT_de‐doped/30_ films revealed temperature‐stable ZT values between 150–298 K, maintaining 0.028 at 298 K (thermal conductivity k = 108 W m^−1^ K^−1^) and 0.021 at 150 K (k = 70 W m^−1^ K^−1^) with <25% variation. Experimental limitation precluded thermal measurements below 150 K and electrical characterization under 100 K in our lab, warranting future studies to probe sub‐100 K thermal transport dynamics in this unique carbon‐based architecture. It can be seen from the subsequent COMSOL simulations in the article (Figure S24, Supporting Information), for TEG modules with a length exceeding 3.0 mm, the temperature difference between the planar structure and the rolled structure can be maintained at low temperatures. Figure 3c shows that ZT of the p‐type CNT film slightly decreased with the temperature. As the ZT of the p‐type CNT at 150 K was ≈0.02, it is reasonable to predict that the ZT will be <0.02 when the temperature is below 150 K.

The CNT_de‐doped/30_ films showed exceptional cryogenic resilience, maintaining stable electrical properties after 4‐h liquid nitrogen immersion (77 K) with natural warm‐up to room temperature (Figure 3d), showing <5% variation in both electrical conductivity and Seebeck coefficients. The films endured 5 thermal cycles (from 77 K to room temperature and back, illustrated in Figure 3e) with <5% resistance fluctuation (Figure 3f), surviving rapid thermal transitions (30‐min warm‐up periods) and incidental moisture exposure during the temperature changes. This thermal stability was attributed to the low thermal expansion of CNT and the tensely packing structure in the films (198% density increase vs pristine). These results demonstrated a triple environmental resilience (ultralow temperature operation, thermal shock tolerance, and humidity resistance) unmatched in flexible thermoelectric.

The mechanical performance of CNT_de‐doped/30_ films was evaluated under ultralow‐temperature conditions (77 K). Flexibility tests conducted in liquid nitrogen demonstrated exceptional bending stability, with the films enduring 180° bending at a 3 mm curvature diameter (Figure 3g; Figure S15, Supporting Information). The PF of CNT_de‐doped/30_ films varied by only 10% during the bending process. While flexible inorganic material‐based thermoelectric films often exhibit a decrease of more than 10% in PF values after 1000 bending cycles at room temperature. For example, the PFs of HP‐PC‐Cu_2_Se and PEDOT: PSS/Cu_2_Se films decrease by 15% after 1000 bends).^[^ ^50^, ^51^ ^]^ The exceptional flexibility at ultra‐low temperature of CNT films should be mainly due to the low efficient of thermal expansion in the range of 10^−6^ /K which is one tenth of that for traditional inorganic thermoelectric materials.^[^ ^27^ ^]^ Resistance and Seebeck coefficient showed negligible fluctuation after 1000 bending cycles in liquid nitrogen (Figure 3h), confirming mechanical durability at cryogenic temperature. Video evidence (Videos S1 and S2, Supporting Information) further verified the material's flexibility in cryogenic environments. Strength measurements revealed a high tensile strength of 0.93 GPa at 128 K (Figure S16, Supporting Information), showing minimal reduction compared to room‐temperature performance (303 K). Testing below 128 K was limited by instrument capabilities. These results highlight the film's remarkable retention of both flexibility and mechanical strength under extreme cryogenic conditions.

A comparative radar analysis (Figure 3i) positioned CNT_de‐doped/30_ films against leading p‐type thermoelectric materials in five critical ultralow‐temperature metrics: electrical conductivity, power factor, density, flexibility, and mechanical strength.^[^ ^52^, ^53^, ^54^, ^55^, ^56^ ^]^ These films demonstrated superior multifunctionality as light‐weight systems, achieving balanced performance across both core thermoelectric properties and environmental resilience. This comprehensive performance profile suggests strong potential for manufacturing integrated thermoelectric generators deployable in polar expeditions or deep‐space missions where multifunctional material stability is paramount.

### 2.4 Temperature‐Dependent TE Properties of n‐Type CNT Films and Rollable TEG Fabricating and Testing

**Figure 4:**
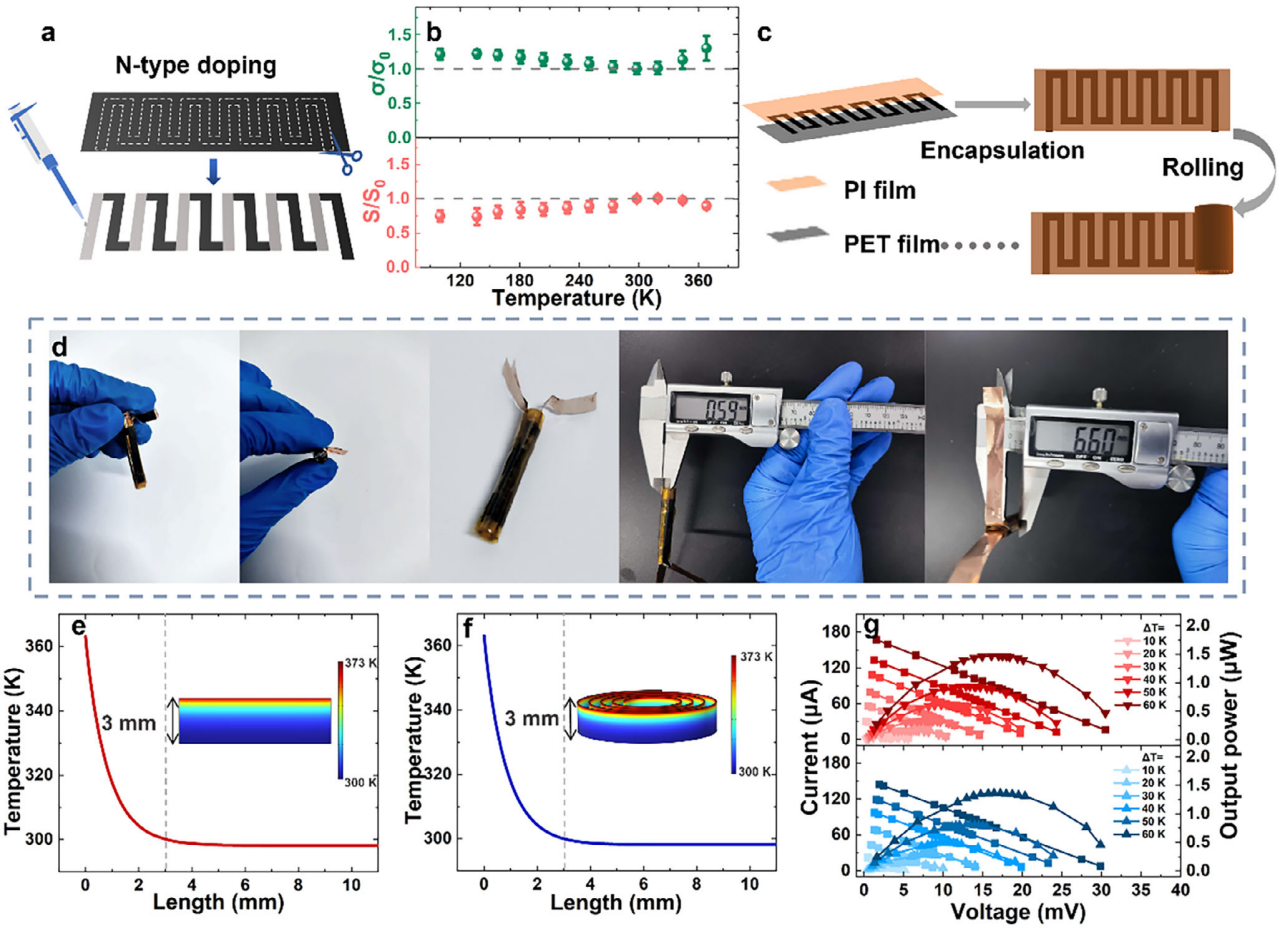
Temperature‐dependent TE properties of n‐type CNT films and rollable TEG fabricating & testing. a) Selective patterning film and n‐type doping of the CNT_de‐doped/30_ films. b) Temperature‐dependent of σ and S of the CNT_n‐doped_. c) Illustration of encapsulation and rolling of TEG. d) Optical image of one rolled TEG. e) Function of the planar TEG temperature and distance from the hot side and temperature distribution diagram. f) Function of the rolled TEG temperature and distance from the hot side and temperature distribution diagram. g) Voltage‐current curves and voltage‐output power curves of the planar TEG and the rolled TEG at different temperature differences.

The thermoelectric conversion capability of CNT films was successfully demonstrated through the fabrication of single‐piece TEG containing 6 p‐n modules within a CNT_de‐doped/30_ film (**Figure** **4**a). The n‐type modules were created through selective patterning and converting p‐type CNT_de‐doped/30_ film into n‐type using PEI as an n‐type dopant. The screening of PEI doping concentration and time is shown in Figure S17 (Supporting Information). Notably, these PEI‐doped CNT (polyethyleneimine‐doped carbon nanotube) films also maintain excellent air stability and thermal stability. After 180 days of ambient exposure, the fluctuations in both electrical conductivity and Seebeck coefficient do not exceed 10% (Figure S17a, Supporting Information). Additionally, when placed in a heating stage at 86 °C for 8 h, the PEI‐doped CNT films exhibit fluctuations in electrical conductivity and Seebeck coefficient both within 10%(Figure S18b, Supporting Information).

Electrical characterization revealed that the PEI‐doped n‐type CNT films (CNT_n‐doped_) exhibited metal‐like electrical conductivity within the 100–298 K temperature range, decreasing from 4.0 MS m^−1^ at 100 K to 3.3 MS m^−1^ at 298 K. While the Seebeck coefficient displayed an inverse temperature dependence, increasing from −30 µV K^−1^ at 100 K to −40 µV K^−1^ at 298 K across the same range. At elevated temperature (298‐368 K), the CNT_n‐doped_ films showed contrasting behavior: electrical conductivity increased from 3.3 MS m^−1^ at 298 K to 4.0 MS m^−1^ at 368 K, while the Seebeck coefficient decreased from −40 µV K^−1^ at 298 K to −36 µV K^−1^ at 368 K. This temperature‐dependent variation in electrical properties at elevated temperature agreed with previous studies,^[^ ^58^, ^59^, ^60^ ^]^ primarily attributed to changes in doping efficiency. Significantly, the CNT_n‐doped_ films maintained a high n‐type PF of 3.6 mW m^−1^ K^−2^ at 100 K, approaching the value of 8.3 mW m^−1^ K^−2^ observed for CNT_de‐doped/30_ films at the same temperature. This comparable performance underscores the exceptional low‐temperature tolerance of CNT_n‐doped_ films, whose PF is 3.6 mW m^−1^ K^−2^, to inorganic materials, such as 3.1 mW m^−1^ K^−2^ for Cu_0.9_Ni_0.1_AgSe,^[^ ^61^ ^]^ 1.4 mW m^−1^ K^−2^ for Mg_3.2_Bi_2–x_Te_x_^[^ ^62^ ^]^ etc.

The single‐piece TEG was subsequently encapsulated using the PET films and PI films substrates (Figure 4c), forming a planar structure that could be rolled into a cylinder configuration with a diameter of 5.42 mm and a thickness of 0.59 mm (Figure 4d). This structural transformation demonstrated the exceptional flexibility inherent to the CNT‐based device. Both planar and cylindrical configurations were evaluated for their thermoelectric conversion capabilities as illustrated in Figure S19 (Supporting Information).

Notably, despite the CNT films’ high thermal conductivity (up to 108 W m^−1^ K^−1^), significant temperature differences between the device's hot and cold sides could be maintained. This thermal stability arises from the substrate‐dominated heat transfer characteristics, the PET films and PI films substrates, being substantially over 250 times thicker (≈90.5 µm) than the CNT films and processing a low thermal conductivity of 0.11–0.15 W m^−1^ K^−1^,^[^ ^63^ ^]^ primarily governed the overall thermal behavior of the TEG modules. The calculation model of the thermal conductivity of the device is shown in the supporting Information.

COMOSOL simulations revealed the temperature distribution profiles for both configurations when subjected to a hot‐side temperature of 363 K (Figure 4e). For TEG modules longer than 3.0 mm, the cold‐side temperature nearly equilibrated with ambient conditions (298 K) in both planar and rolled configurations, with detailed simulation methodology provided in the Supporting Information. The planar TEG was fixed on a heating plate at 100 °C, while the rolled TEG was placed on the 100 °C thermal paste. The infrared thermal image results showed that both structures could maintain a good temperature difference (as shown in Figure S20, Supporting Information). The planar and cylindrical TEGs exhibited comparable output open‐circuit voltage (V_oc_), current and power output under equivalent temperature differences (Figure 4g), with more thermoelectric characterization data presented in Figure S21 (Supporting Information).

### 2.5 Cryogenic Temperature Tolerance of n‐Type CNT Films and Planar TEG

**Figure 5:**
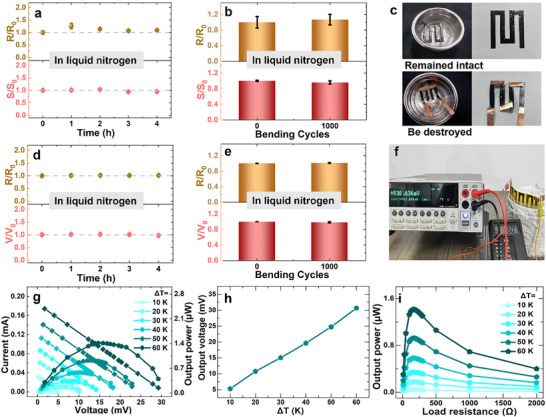
Cryogenic temperature tolerance of n‐type CNT films and planar TEG a) The resistance and Seebeck coefficient of the CNT_n‐doped_ films as a function of soaking time in liquid nitrogen. b) The resistance and Seebeck coefficient varied of the CNT_n‐doped_ films after bending in liquid nitrogen. c) Optical images of changes in single‐piece and non‐single‐piece TEG with 2 pairs soaked in liquid nitrogen for 3 h. d) The resistance and output voltage varied of the single‐piece TEG as a function of soaking time in liquid nitrogen. e) Variations in R and V of the single‐piece TEG in liquid nitrogen as a function of soaking time in liquid nitrogen. f) Optical image of the single‐piece TEG performance tested at low temperature. g) The voltage‐current curves and voltage‐output power curves of the single‐piece TEG at different temperature differences. h) Measured voltage output of the single‐piece TEG at different temperature differences. i) The output power of the single‐piece TEG as a function of load resistance.

The low‐temperature tolerance of the CNT_n‐doped_ films and the single‐piece TEG was systematically evaluated. As demonstrated in **Figure** **5**a,b, the CNT_n‐doped_ films exhibited exceptional electrical and bending stability in liquid nitrogen. Similar to CNT_de‐doped/30_ films, the CNT_n‐doped_ films showed minimal variation (less than 5%) in both electrical conductivity and Seebeck coefficients while being stored in liquid nitrogen for ≈4 h. Furthermore, after 1000 bending cycles in the liquid nitrogen, the CNT_n‐doped_ films retained nearly consistent thermoelectric performance, underscoring their robust mechanical durability under extreme thermal stress.

To validate low‐temperature operational stability, we fabricated a single‐piece TEG with two p‐n module pairs and subjected to cryogenic testing. As shown in Figure 5c, the device maintained structural integrity without observable degradation after full immersion in liquid nitrogen. For comparative analysis, a conventional π‐structure TEG was constructed using copper interconnects and silver paste bonding. This reference device exhibited catastrophic failure after immersing in liquid nitrogen since the metal‐CNT thermal expansion mismatch induced interfacial delamination, resulting in immediate electrical disconnection.

We evaluated the electrical and mechanical stability of the single‐piece TEG under cryogenic conditions. As shown in Figure 5d, the device maintained stable electrical performance in liquid nitrogen immersion tests, with less than 5% variation in both resistance and output voltage during prolonged 4‐h exposure. Remarkably, even after 1000 bending cycles in liquid nitrogen (Figure 5e), the TEG retained over nearly consistent its thermoelectric performance. This contact resistance of the single‐piece TEG composed of 6 p‐n modules was roughly calculated by the difference between the theoretical resistance and the experimental resistance according to literature,^[^ ^64^ ^]^ which accounts for ≈13.3% of the total resistance. This value is approaching to the contact resistance ratio of state‐of‐the‐art optimized inorganic thermoelectric devices (≈9.8%).^[^ ^65^ ^]^ Moreover, the contact resistance ratio did not increase after 1000 bending cycles in the liquid nitrogen as shown in Figure S22 (Supporting Information). This exceptional low‐temperature durability originates from the matched thermal expansion properties and robust interfacial boning between the p‐type and n‐type CNT films. The results conclusively demonstrate superior cryogenic stability of our single‐piece TEG compared to conventional π‐structure devices, which typically fail under similar thermal‐mechanical stress.

The heat‐to‐electricity conversion capability of the single‐piece TEG was evaluated using a device with six p‐n module pairs (Figure 5f). In this configuration, the hot side of the TEG was maintained at ambient temperature (≈25 °C), while the cold side was close to liquid nitrogen in the container. By vertically adjusting the device's position relative to the liquid nitrogen surface, we precisely controlled the temperature difference (∆T) across the TEG. Both the output voltage and current value exhibited linear increases with ∆T, reaching 30.6 mV and 0.174 mA, respectively at ∆T = 60 K (Figure 5g,h). Maximum output power of 1.41 µW was achieved under matched load conditions (R_load_ = 166 Ω, equals to the device's internal resistance) at ∆T = 60 K (Figure 5i). The TEG conversion efficiency at cryogenic temperatures (Figure S23, Supporting Information) demonstrated parity with performance observed in conventional near‐room‐temperature operation (Figure S21c, Supporting Information), highlight the device's broad operational viability across extreme thermal difference. The COMOSOL simulations revealed the temperature distribution profiles for both configurations when subjected to a cold‐side temperature of 77 K (Figure S24, Supporting Information).

The water tolerance property is important as water droplets will condense on the device when using the TEGs at cold environments. Figure S25a (Supporting Information) showed that p‐type CNT_de‐dedoped/30_ films exhibited an excellent water tolerance in terms of their Seebeck coefficients which maintained ≈100% of the initial values after the films were kept in water for over 14 days. While the electrical conductivity of p‐type CNT_de‐dedoped/30_ films decreased a little after 2‐day storage in water and then became nearly constant while prolong the storing time up to 14 days. The water‐tolerant electrical conductivity and See beck coefficient resulted in 90% of the initial PF of the p‐type CNT_de‐dedoped/30_ films after it were kept in water for 14 days. Differently, n‐type PEI doped CNT films exhibited an obvious degradation in water, which maintained only 60% of their initial PF value after being kept in water for 14 days (Figure S25b, Supporting Information). These results are consistent with previous literature.^[^ ^37^, ^66^ ^]^ To improve water tolerance, the single‐piece TEG was encapsulated with polyethylene terephthalate (PET) and polyimide (PI) films as shown in Figure 4c in the original manuscript. Figure S25c (Supporting Information) showed that the encapsulated single‐piece TEG maintained nearly constant of its output power while the non‐encapsulated single‐piece TEG degraded obviously after being kept in water for 14 days.

### 2.6 Fabrication and Performance Measurement of the Mini TE Power Station

**Figure 6:**
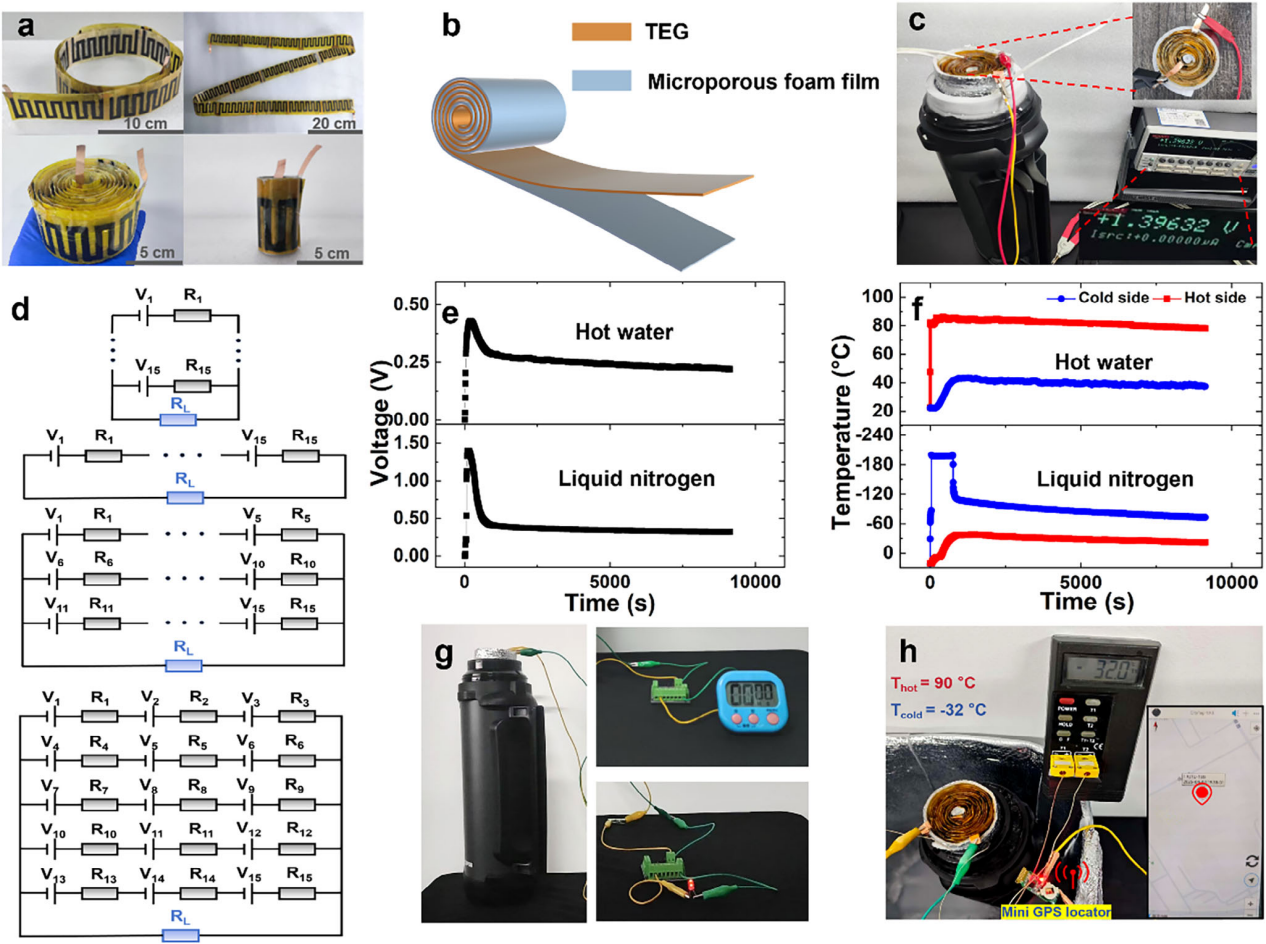
Fabrication and performance measurement of the mini TE power station. a) Optical images of the integrated TEG. b) Schematic diagram of integrated flexible TEG and microporous foam film. c) Optical image of output voltage measurement when the cold side is cooled by liquid nitrogen. d) Circuit diagram of an integrated TEG system with 15 thermoelectric modules operating at maximum output power. e) Time‐dependent output voltage of the rolled integrated TEG when the cup is filled with hot water or liquid nitrogen. f) Temperature variations over time at the hot and cold sides of the rolled integrated TEG under hot water or liquid nitrogen conditions. g) Optical images of the mini thermoelectric power station powering a red LED and an electronic timer. h) When the ambient temperature is −32 ° C, the mini thermal power station activates the mini GPS locator when the cup is filled with hot water.

To assess long‐term power generation capabilities, we developed a prototype thermoelectric power station by integrating a single‐piece CNT‐based TEG (90 p‐n module pairs) with a 2 L thermos cup serving as a phase‐change thermal reservoir. Additional experiments have been performed to test the output power of TEGs with 30‐pair and 60‐pair modules under the same conditions (ΔT = 220 K). The output powers of the TEGs with 30‐pair and 60‐pair modules are 64 and 124 µW, respectively (Figure S26, Supporting Information). The contact resistance ratios increase slightly with the module number which is 14%, 16%, and 17% for TEGs with 30‐pair, 60‐pair and 90‐pair modules, respectively. No obvious non‐linear attenuation was observed in Figure S26 (Supporting Information). The slightly increase of the contact resistance ratios with the module number should be due to manual error. This modest performance reduction has also been observed in previous works while increasing the module numbers.^[^ ^67^ ^]^ As illustrated in **Figure** **6**a, the flexible TEG (length: 180 cm) was rolled into a cylindrical configuration (diameter: 6.5 cm), maintain mechanical integrity across bending radii. For enhanced thermal insulation, the rolled TEG was encapsulated with a microporous foam film (aluminum laminated sponge, Figure 6b) to minimize parasitic heat transfer. Under steady‐state operation with liquid nitrogen (−196 °C) in the reservoir and ambient air exposure (≈25 °C) on the hot side, the system generated a sustained voltage of 1.40 V (Figure 6c). Output optimization through impedance matching was demonstrated via programmable series‐parallel circuit configuration (Figure 6d). Theoretical modeling at ∆T = 60 K (Table S2, Supporting Information) predicts tunable generated voltage and current through adaptive circuit reconfiguration, enabling precise alignment with real‐time load demands in practical applications.

We characterized the operation durability of the mini thermoelectric power station under extreme thermal conditions. As shown in Figure 6e, the system generated initial voltages 0.43 V (hot water at 86 °C, ∆T = 60 K) and 1.40 V (liquid nitrogen at −196 °C, ∆T = 220 K), respectively. Both configurations exhibited rapid voltage decay during the initial ∆T equilibration phase (Figure 6f), stabilizing at sustained outputs of 0.25 V (hot water) and 0.32 V (liquid nitrogen) over 2.5 h operation. Notably, the cryogenically power station (∆T = 221 K) successfully drove low‐power electronics with an amplifier, including a digital timer and an LED (Figure 6g). This design circuit (Figure S27, Supporting Information) does not require an additional power supply, but the using of booster (model STEVAL‐ISV019V1) causes ≈60% energy loss. In a simulated polar environment (−32.0 °C ambient), the hot‐water configuration (∆T = 122.5 K) demonstrated a promising way to operate a miniature GPS locator (90.5 °C thermal reservoir, Figure 6h). The purpose of the GPS locator in Figure 6h is only to validate the concept's feasibility. The schematic diagram of the circuit was shown in Figure S28 (Supporting Information). The model of the voltage amplifier is ADA4528 with a rated power of 0.4 W. The rated power of of the GPS locator is ≈2 mW. Additional power supply is required to support the GPS locator and the amplifier as the mini power station. However, when increase the number of the CNT modules to 1300‐pair, the mini power station can offer sufficient power to supply the GPS locator alone assuming the contact resistance ratio is 20% as shown in Figure S29 (Supporting Information). As the purpose of the GPS locator in Figure 6h is only to validate the concept's feasibility, it may not be necessary to have a mini power station with 1300‐pair CNT modules since it is very time‐consuming to fabricate. Amplifiers were also often used in previous works to demonstrate the potential application feasibility of power‐ generating devices.^[^ ^68^, ^69^, ^70^, ^71^ ^]^ Further performance scaling can be achieved through two synergistic strategies: increasing p‐n module number and implementing vacuum‐sealed aerogel barriers to extend ∆T duration.

## 3 Conclusion

In this work, we developed thermally annealed CNT films with tunable doping levels that achieve exceptional thermoelectric performance across extreme temperature ranges. The lightweight CNT films (1.16 g cm^−3^) exhibited a high room temperature p‐type power factor of 10.5 mW m^−1^ K^−2^, maintaining 8.3 mW m^−1^ K^−2^ at cryogenic temperatures (100 K) while demonstrating remarkable mechanical flexibility and robustness even under −196 °C conditions.

We further demonstrated practical applications through a rollable single‐piece thermoelectric generator featuring six p‐n modules created by selective PEI doping. Encapsulated with low‐thermal‐conductivity PET/PI substrates, the compact TEG achieved equivalent output power in both planar and rolled configurations across near‐room‐temperature and cryogenic environments. By integrating this TEG with thermal storage tanks and adaptive circuitry, we constructed a mini thermoelectric power station capable of generating volt‐scale electrical outputs of 0.42 V (∆T = 60 K)‐1.4 V (∆T = 220 K) and delivered high output powers in 14 µW (∆T = 60 K)‐175 µW (∆T = 220 K) range using either hot water (≈86 °C) or liquid nitrogen (−196 °C) as thermal storage media, sustaining operation for >2.5 h to power small electronics like timers, LEDs and GPS devices. This work establishes a scalable paradigm for flexible energy harvesting, bridging high‐performance material engineering (through doping optimization and structural design) with sustainable applications that exploit both industrial waste heat and cryogenic thermal differences. The combination of temperature‐agnostic performance, mechanical durability, and roll‐to‐roll process compatibility positions CNT films as promising candidates for future flexible thermoelectric devices.

## 4 Experimental Section

### Materials

Ethanol was purchased from Tianjin Fuyu Fine Chemical Co., Ltd., China. Methanol was purchased from Sinopham Chemical Reagent Co., Ltd., China. N‐hexane was purchased from Tianjin Zhiyuan Chemical Co., Ltd., China. Thiophene and ferrocene were Meryer Chemical Technology Co., Ltd., China. Hydrochloric acid (HCl) was purchased from Kelong Chemical Co., Ltd., China. Sulfurochloridic acid (CSA) was purchased from Meryer Chemical Technology Co., Ltd., China. Polyethyleneimine (PEI) (99%) was purchased from Macklin Co., Ltd., China. Dimethyl sulfoxide (DMSO) was purchased from Energy Chemical Co., Ltd., China. All the chemicals were used as received.

### Synthesis of CNT Aerogel

The CNT aerogel was synthesized in a horizontal furnace maintained at 1300–1500 °C using nitrogen (N_2_) as the carrier gas. The precursor solution was prepared by dissolving ferrocene and thiophene in a mixture of n‐hexane and ethanol, followed by 30‐min ultrasonic treatment. This homogeneous solution was subsequently injected into the reactor at a controlled rate of 1–2 mL min^−1^. The precursor‐laden nitrogen flow (0.5–1 L min^−1^) then transported the mixture into the high‐temperature zone of the furnace. Finally, the synthesized aerogel was ejected from the reactor into ambient atmosphere using nitrogen and collected by a rotating roller.

### Preparation of CNT_as‐syn_ Film

A 10‐cm‐diameter winding drum was employed for CNT aerogel collection. The drum surface was covered with sulfate paper substrate. During the preparation process, the winding rate was controlled at 3 mm s^−1^. Throughout the collection phase, ethanol mist was periodically sprayed onto the forming film. After 30 min of continuous collection, the composite film was carefully peeled from the drum. Subsequently, the film underwent compression treatment using a hydraulic press (Kejing MSK‐2150, China) under 4‐ton force for 5 min to achieve smooth and densely packed surface morphology.

### Treatment of CNT_as‐syn_ Film

The obtained CNT_as‐syn_ films were first annealed in a tube furnace under inert atmosphere at 1273 K for 16 h. Subsequently, the thermally treated samples were immersed in concentrated HCl for 2 h (CNT_HCl_). The acid‐washed films underwent triple rinsing cycles with deionized water, followed by ambient air‐drying at room temperature. Finally, the dried films were subjected to CSA treatment at 423 K for densification, resulting in acid‐doped CNT membranes.

### De‐Doping of CNT_acid‐doped_ Film

The CNT_acid‐doped_ films were heat treated in a horizontal furnace under flowing N_2_ (0.01 L min^−1^). The dedoping of the system is studied under the control of time and temperature parameters.

### N‐Type Doping Method

The PEI dopants were dissolved in DMSO at certain concentrations by weight. The n‐type doping process involves depositing a controlled amount of PEI solution onto the CNT_de‐doped/30_ film using a micropipette, followed by ambient‐pressure drying on a 100 °C hot plate. After a specific heating duration, the dopant molecules are deposited onto the CNT films' surface.

### Preparation of the Single‐Piece TEG

The single‐piece TEG with 6 pairs of p‐n legs were fabricated by hand. The length and width of the TE leg are 35 mm and 5 mm, respectively. Then 3 wt.% PEI solution was alternately dropwise to the thermoelectric legs in series for doping, the residual liquid was sucked by using filter paper and on a 100 °C hot plate. The PET films and PI films were then used for plastic encapsulation to obtain one single piece TEG, which was a plane structure. The single‐piece TEG could be rolled into a cylinder configuration with a diameter of 5.42 mm and a thickness of 0.59 mm.

### Preparation of the Mini Thermoelectric Power Station

The mini thermoelectric power station is constructed by integrating 15 single‐piece CNT‐based TEGs containing 90 p‐n module pairs, with a 2 L thermos cup serving as a phase‐change thermal reservoir. The flexible TEG (length: 180 cm) is rolled into a rolled configuration (diameter: 6.5 cm). The TEG assembly is electrically connected in series using silver paste and reinforced with adhesive tape. To enhance thermal insulation, the rolled TEG is encapsulated with a microporous foam film (aluminum‐laminated sponge), effectively minimizing parasitic heat transfer.

### Theoretical Carrier Weighted Mobility Value Model

The weighted mobility was calculated according to literature,^[^ ^37^ ^]^ which revealed that the decrease of the carrier concentration should be mainly responsible for the quick change of the electrical conductivity.

 literature with the following equation:

(3) $$\begin{array}{ccc} \mu_{W} & = & \frac{3h^{3}\sigma }{8\pi e{\left(2m_{e}k_{B}T\right)}^{3/2}}\;\left[\frac{\exp\left[\frac{\left|S\right|}{k_{B}/e}-2\right]}{1+\exp\left[-5\left(\frac{\left|S\right|}{k_{B}/e}-1\right)\right]}\right.\\ & & \left.+\;\frac{\frac{3}{\pi^{2}}\frac{\left|S\right|}{k_{B}/e}}{1+\exp\left[5\left(\frac{\left|S\right|}{k_{B}/e}-1\right)\right]}\right] \end{array}$$

where µ_w_ is the weighted mobility, h is the Planck constant, me is the electron mass, T is the absolute temperature in K, S is the Seebeck coefficient, and k_B_/e = 86.3 µV K^−1^. The weighted mobility values were 160, 2417 and 2109 cm^2^ V^−1^ S^−1^ for CNT_as‐syn_, CNT_acid‐doped_ and CNT_de‐doped/30_ films, respectively. The CNT_acid‐doped_ films had a similar weight mobility compared to the reported densified CNT films in literature.^[^ ^37^, ^72^ ^]^ The sharp increase of the weighted mobility from 160 to 2417 cm^2^ V^−1^ S^−1^ resulted in the significant improvement of the electrical conductivity of the CNT_acid‐doped_ films, which was attributed to the densification and doping during the treatment process. Although the weight mobility decreased a little from 2417 to 2109 cm^2^ V^−1^ S^−1^, the electrical conductivity decreased quickly from 11.7 MS m^−1^ for CNT_acid‐doped_ films to 2.8 MS m^−1^ for CNT_de‐doped/30_ films. The results revealed that the decrease of the carrier concentration should be mainly responsible for the quick change of the electrical conductivity during the de‐doping process.

### Density Calculation Method

The practical density of CNT films could be calculated with the equation: density = mass/volume. The mass of the sample was obtained with a high‐precision microbalance and the volume of the sample was estimated by the following equation: volume = length x width x thickness.

### Theoretical Calculation of the Thermal Conductivity of Devices

The thermal conductivity of the TEG device after packaging is calculated using parallel model,^[^ ^73^ ^]^ and the formula is as follows:

(4) $$\frac{1}{\kappa_{T}}=\frac{1-\phi_{y}-\phi_{z}}{\kappa_{x}}+\frac{\phi_{y}}{\kappa_{y}}+\frac{\phi_{z}}{\kappa_{z}}$$

where κ_*T*_ represents the total thermal conductivity; κ_*x*_ represents the thermal conductivity of the carbon nanotube film; κ_*y*_ and κ_*z*_ represent respectively the thermal conductivity of the PET films and PI films substrates; ϕ_*y*_ and ϕ_*z*_ represent respectively the volume fraction of the PET films and PI films substrates.

### Characterization

The Seebeck coefficient and electrical conductivity were measured using the commercial characterization systems (NETZSCH SBA‐458, Germany, JouleYacht MRS‐3, China). Electrical conductivity was determined via the four‐point probe method, while the Seebeck coefficient was simultaneously acquired through dynamic heating/cooling measurements. Thermal conductivity characterization was conducted using a separate commercial instrument (ZHONGXIN VTET‐01, China). Three or more samples were tested to get each data point. The error (E) was calculated according to the equation:

(5) $$E=\sqrt{\sum_{i=1}^{n}{\left(X_{i}-X_{m}\right)}^{2}/n}\;$$

and

(6) $$X_{m}=\sum_{i=1}^{n}X_{i}/n\;$$

where 𝑋_𝑖_ is experimental data and n is the number of samples, respectively.^[^ ^74^, ^75^ ^]^

Scanning electron microscope (SEM) images were obtained using FEI QUANTA 250 FEG, USA. Transmission electron microscope (TEM) images were obtained with a JEOL 2010D, Japan, accelerating voltage, 200 kV. Thermogravimetric analyses (TGA) of MWCNT films were performed under the air atmosphere using a synchronous Thermal Analyzer Q600, USA, with a heating rate of 10 °C min^−1^. X‐ray photoelectron spectroscopy (XPS) was performed with ThermoFisher Scientific ESCALAB Xi+, USA. Raman spectra were recorded with a ThermoFisher Raman spectrometer, USA, with an excitation wavelength of 514 nm. as shown in the supplementary information. The output voltage of Single‐TEG was obtained using a Keithley 2400 Multimeter, USA. The CNT samples were cut into strips with an aspect ratio of 2 (a length of 10 mm and a width of 5 mm). And their mechanical properties were tested by DMA850 (TA Instruments, USA).

## Conflict of Interest

The authors declare no conflict of interest.

## Supporting information



Supporting Information

Supplemental Video 1

Supplemental Video 2

## Data Availability

The data that support the findings of this study are available from the corresponding author upon reasonable request.
