# Supplementary material for: Rollable Single‐Piece Thermoelectric Generators at Cryogenic Temperature Fabricated with High‐performance CNT Films Achieved by Doping Modulation
Source: Adv Sci (Weinh). 2025 Oct 21;13(1):e15688. doi: 10.1002/advs.202515688 (PMC12767057; doi:10.1002/advs.202515688)
Supplement: Supplementary file 1 — Supporting Information [file ADVS-13-e15688-s001.docx]

Supporting information

Rollable single-piece thermoelectric generators at cryogenic temperature fabricated with high-performance CNT films achieved by doping modulation

Zihan Zhu^1^, Kuncai Li^1^, Xin Hao^3^, Xu Dai^1^, Jing Wang^2^, Fenfen Yin^4^, Hong Wang^1,2,3^*

^1^Frontier Institute of Science and Technology, Xi’an Jiaotong University, Xi’an, 710054, China

^2^State Key Laboratory of Multiphase Flow in Power Engineering, Xi’an Jiaotong University, Xi’an, 710054, China

^3^School of Energy and Power Engineering, Xi’an Jiaotong University, Xi’an, 710054, China

^4^Shaanxi Canon Flexible Thermoelectric Technology Co., Ltd, Xi’an, Shaanxi Province, 710000, China

E-mail: [hong.wang@xjtu.edu.cn](mailto:hong.wang@xjtu.edu.cn)


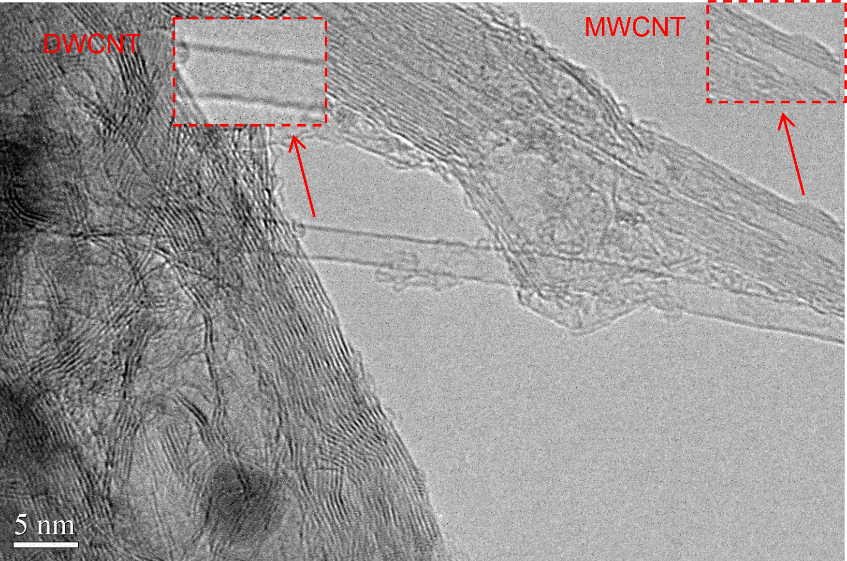


Figure S1. TEM image of the CNT_as-syn_ films.


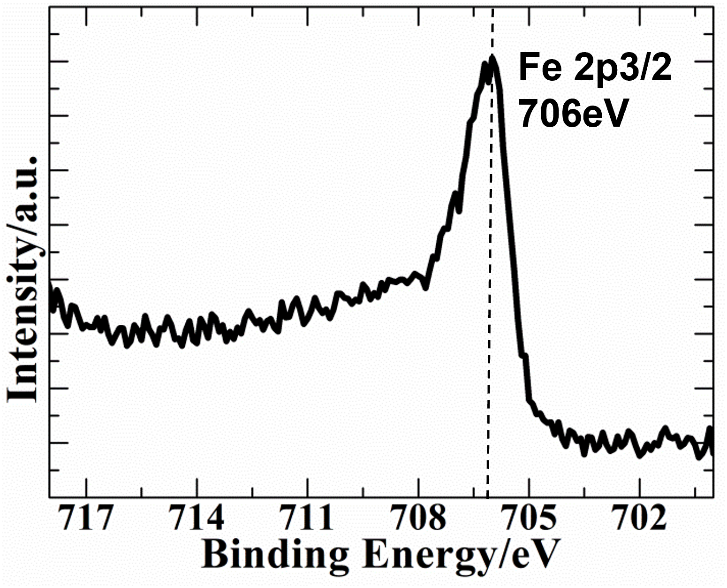


Figure S2. XPS spectrum of the CNT_as-syn_ films.

The XPS spectrum shows a peak near 706 eV, which is assigned to iron or iron carbide nanoparticles^[1]^.


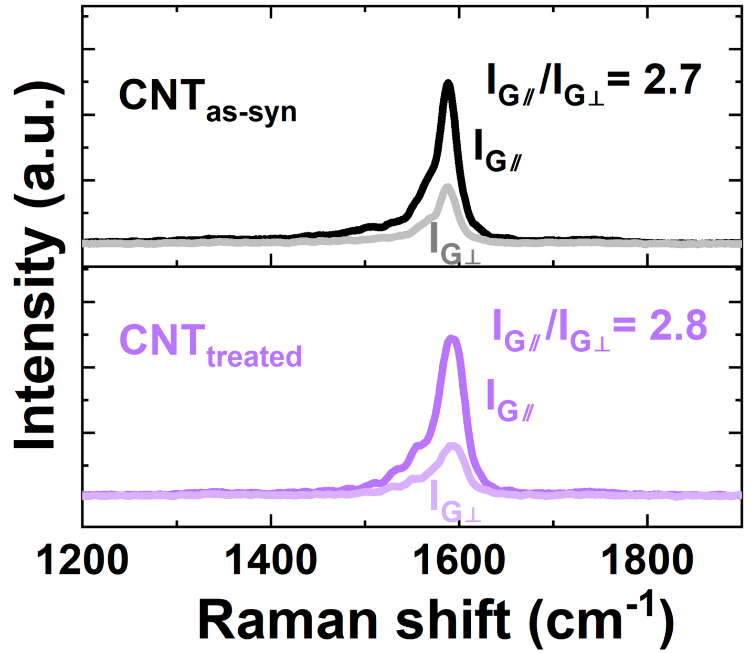


Figure S3. Raman spectra of the CNT_as-syn_ films and the CNT_acid-doped_ films.

The CNT_as-syn_ films and the CNT_acid-doped_ films are proved to be anisotropic by polarized Raman spectroscopy. The G-band intensity of CNT_as-syn_ films in the direction parallel to the rolling direction (I_G//_) is about 2.7 times higher than that in the perpendicular direction (I_G⊥_), while the I_G//_: I_G⊥_ in CNT_acid-doped_ films is 2.8.


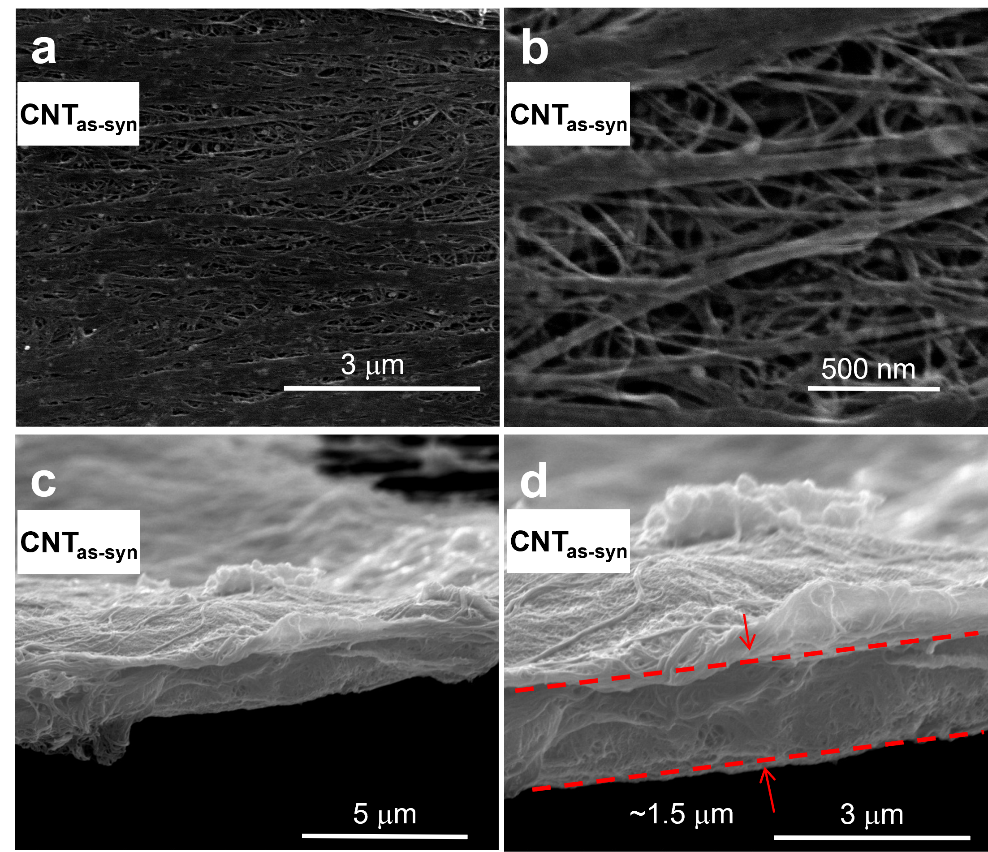


Figure S4. SEM images of the morphology (a)-(b) and the cross-section (c)-(d) of the CNT_as-syn_ films.

The thickness of CNT_as-syn_ films is identified to be ~1.5 μm. The surface of the CNT_as-syn_ films is porous with holes at the diameter of hundreds of nanometers.


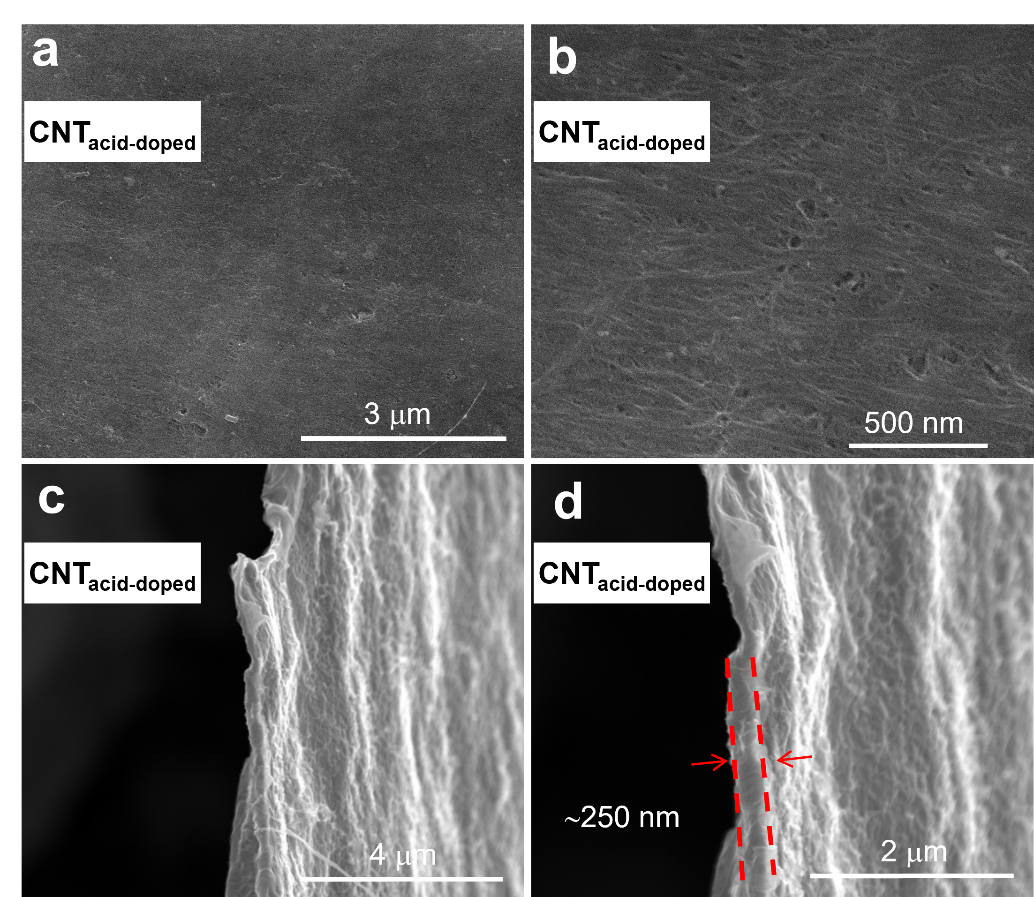


Figure S5. SEM images of the morphology (a)-(b) and cross-section (c)-(d) of the CNT_acid-doed_ films.

The thickness of the CNT_acid-doped_ films is ~250 nm. The surface of the CNT_acid-doped_ films much smoother and tight compared to the surface of CNT_as-syn_ films. These morphological improvements align with previously reported doping-induced structural optimization in carbon nanotube networks ^[2]^.


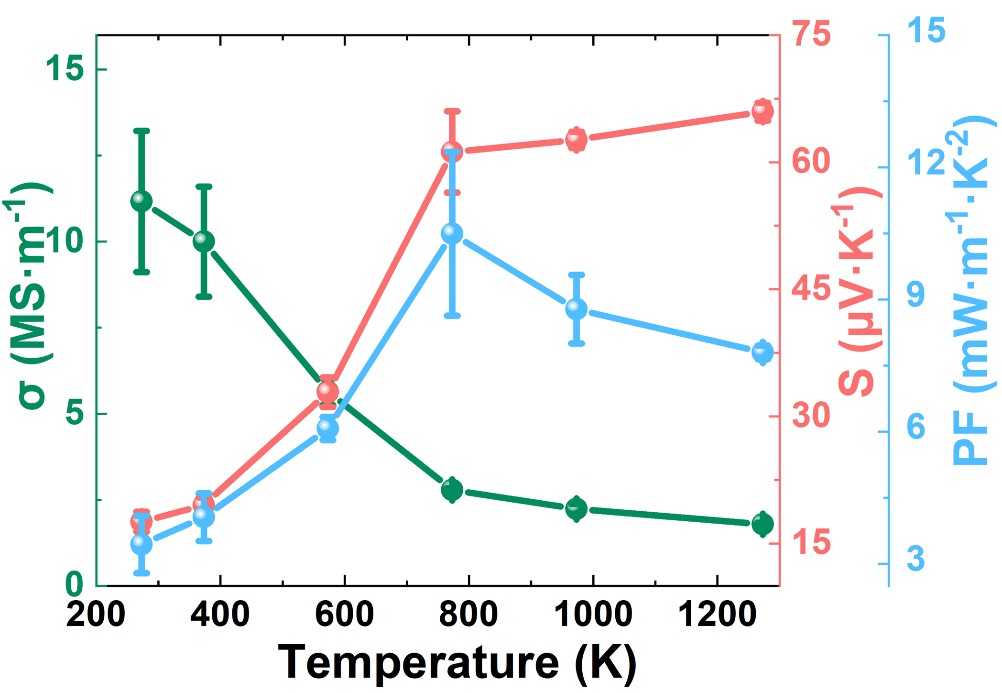


Figure S6. The σ, S and PF of the CNT_acid-doped_ films varied with the de-doping temperature at the time of 30 min.


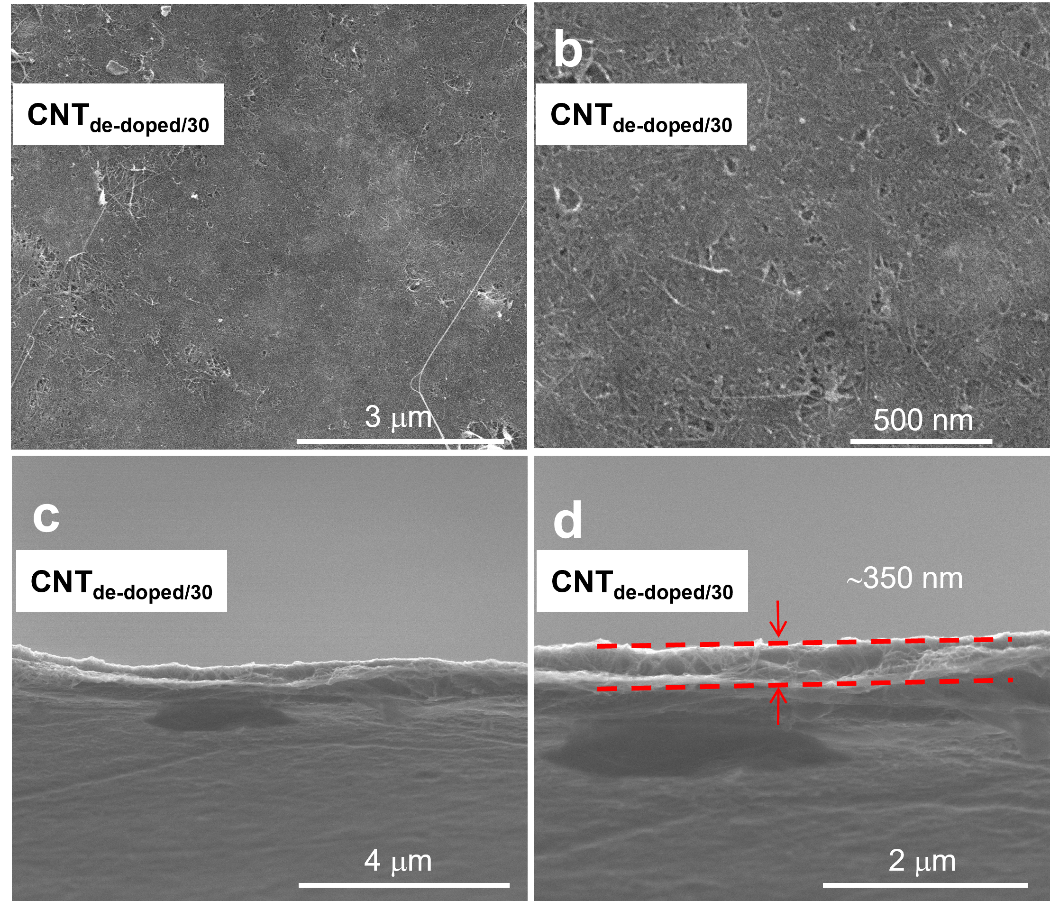


Figure S7. SEM images of the morphology (a)-(b) and cross-section (c)-(d) of the CNT_de-doped/30_ films.

The thickness of the CNT_de-doped/30_ films is ~350 nm. It can be seen from the image that the surface of the film is relatively smooth.


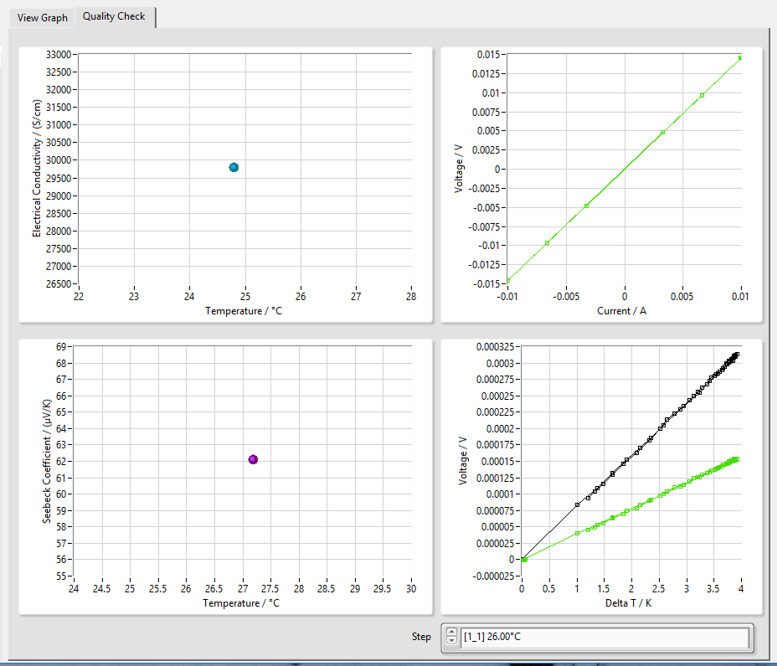


Figure S8. The Screenshots during the electrical conductivity and Seebeck coefficient tests.


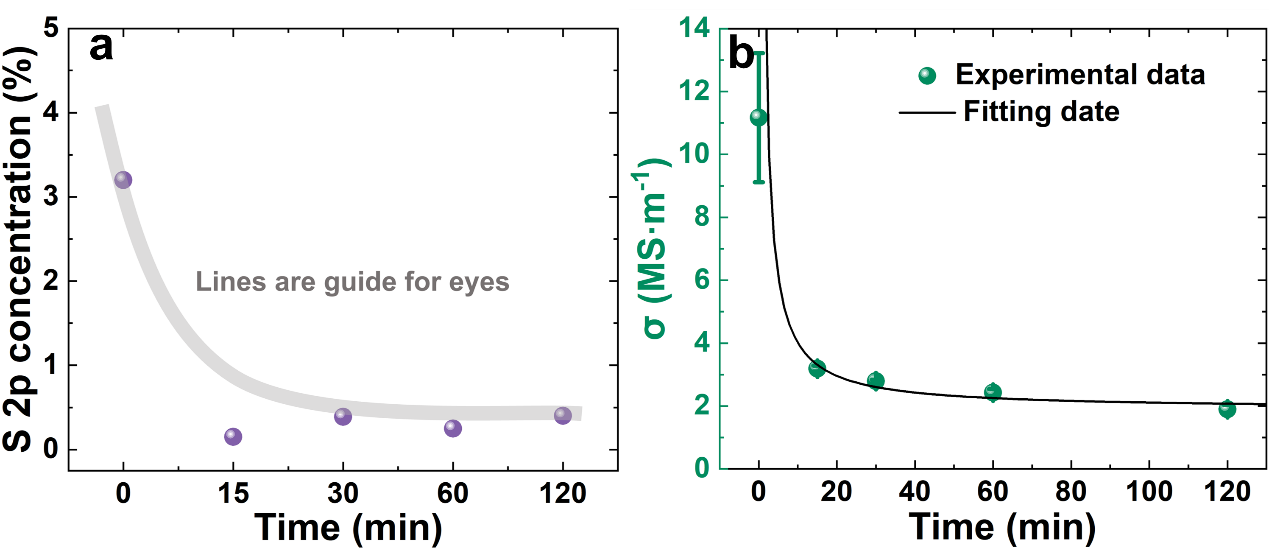


Figure S9. (a) The S 2p concentration of CNT_acid-doped_ films varied with the de-doping time. (b) σ and fitting results by pseudo-second-order model of CNT_acid-doped_ films varied with the de-doping time.


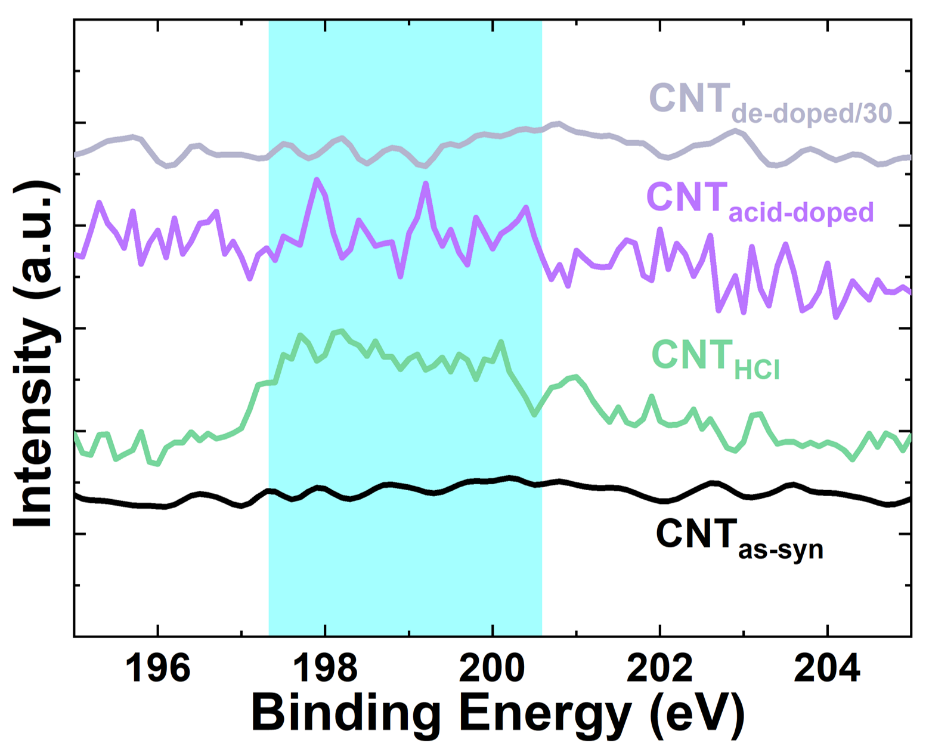


Figure S10. The XPS spectra of Cl2p for the CNT_as-syn_, the CNT_HCl_, the CNT_acid-doped_ and the CNT_de-doped/30_ films.


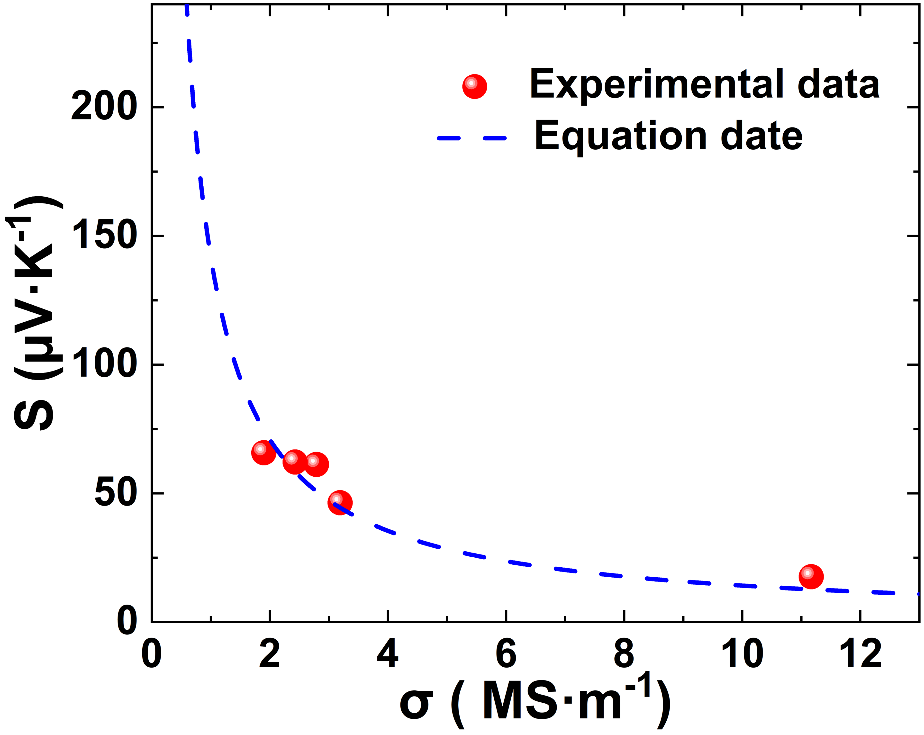


Figure S11. The inverse relationship between the conductivity and Seebeck coefficient of the CNT_acid-doped_ films, CNT_de-doped/15_ films, CNT_de-doped/30_ films, CNT_de-doped/60_ films, CNT_de-doped/120_ films, and the dashed line obtained by the Kang-Snyder equation.

According to the Kang-Snyder Equation, the inverse relationship between $\sigma$ and $\left| S \right|$ could be described as follows:

$$\left| S \right|=\frac{\kappa_{B}}{e}\frac{\pi^{2}}{3}s\left( \frac{\sigma}{\sigma_{E}} \right)^{-1/s}$$

In the Kang-Snyder equation, $\sigma_{E}$ was the temperature-independent but energy-dependent parameter, and *s* was a transport parameter^[3]^.


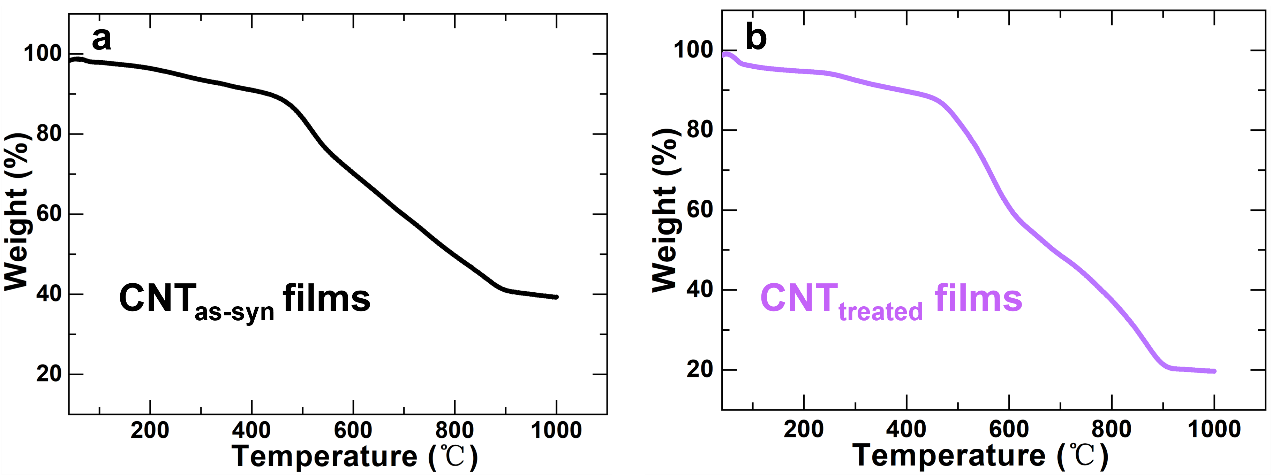


Figure S12. TGA curves of the CNT_as-syn_ films (a) and CNT_acid-doped_ films (b).

The CNT_as-syn_ films loss weight at ~350 °C and ~450 °C. The first weight loss of the original CNTs is attributed to the degradation of amorphous carbon^[4]^. The second weight loss is due to the oxidation of carbon nanotubes^[5]^. after being heat at 1000 °C in the air, there is still about 19 wt.% of mass remained for the CNT_acid-doped_ films, which is mainly composed of iron oxides.


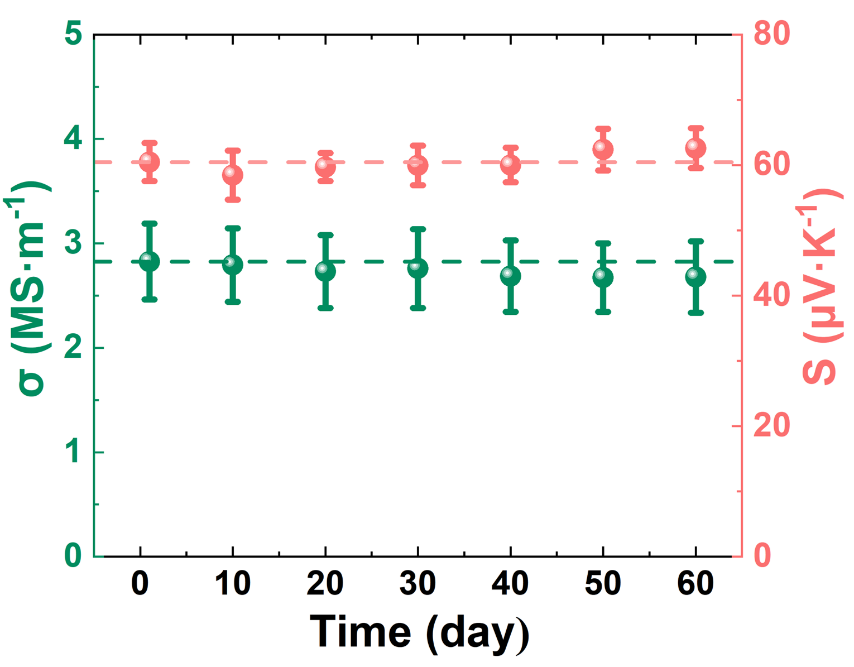


Figure S13. The σ and S of the CNT_de-doped/30_ films as a function of storing time in the air at room temeprature.


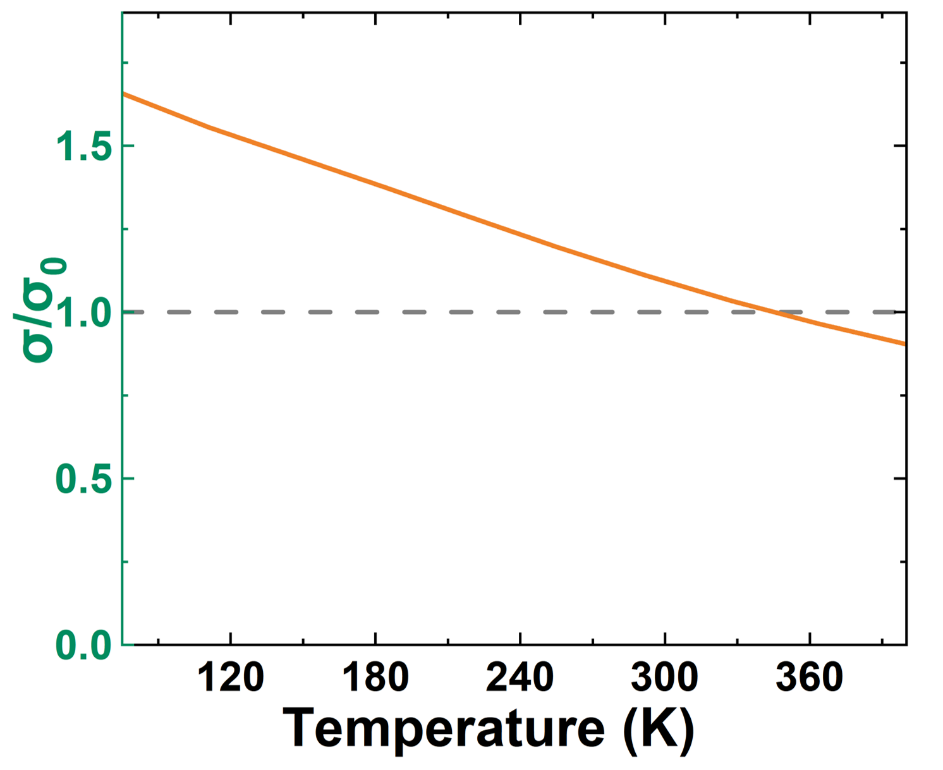


Figure S14. Inverse relationship of the conductivity and the temperature for the CNT_de-doped/30_ films. The orange line is obtained by a heterogeneous model.

The temperature-dependent curve can be well fitted by the following equation^[6]^.

$\rho\left( T \right)=f_{1}\rho_{t}\exp\left( -\frac{T_{t}}{T+T_{s}} \right)+f_{2}\alpha T+f_{3}\rho_{b}exp(-\frac{T_{b}}{T})$

The heterogeneous model has been used to fit the temperature-dependent electrical conductivity by Kaiser et. al. Detailed discussion could be found in previous literature^[7]^.


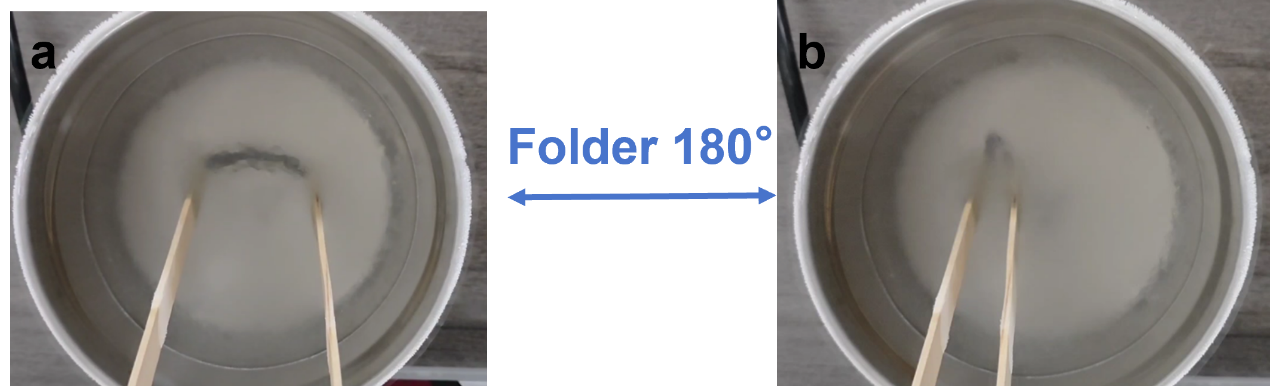


Figure S15.Optical image of the CNT_de-doped /30_ films bent 180°in liquid nitrogen.


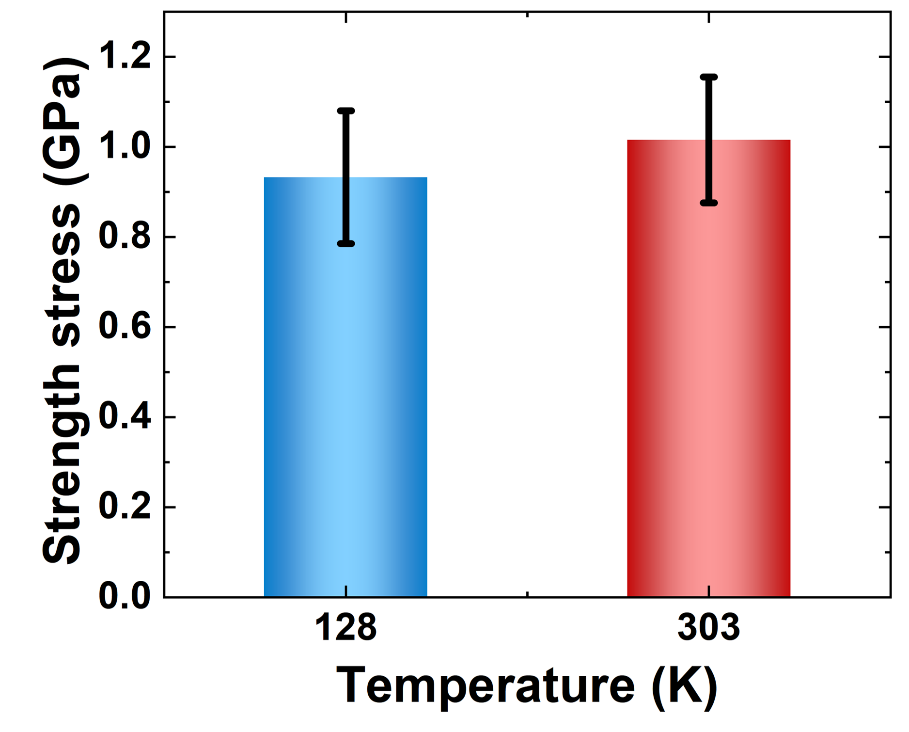


Figure S16. The strength stress values of the CNT_de-doped /30_ films at 128K and 303K.


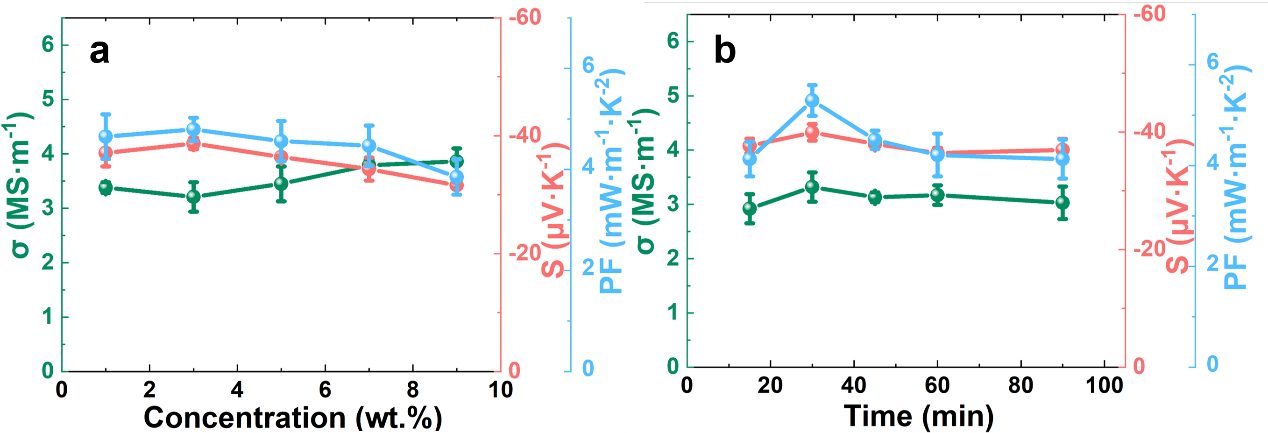


Figure S17. The σ, S and PF of the CNT_n-doped_ films varied with the concentration of PEI and the treatment time.


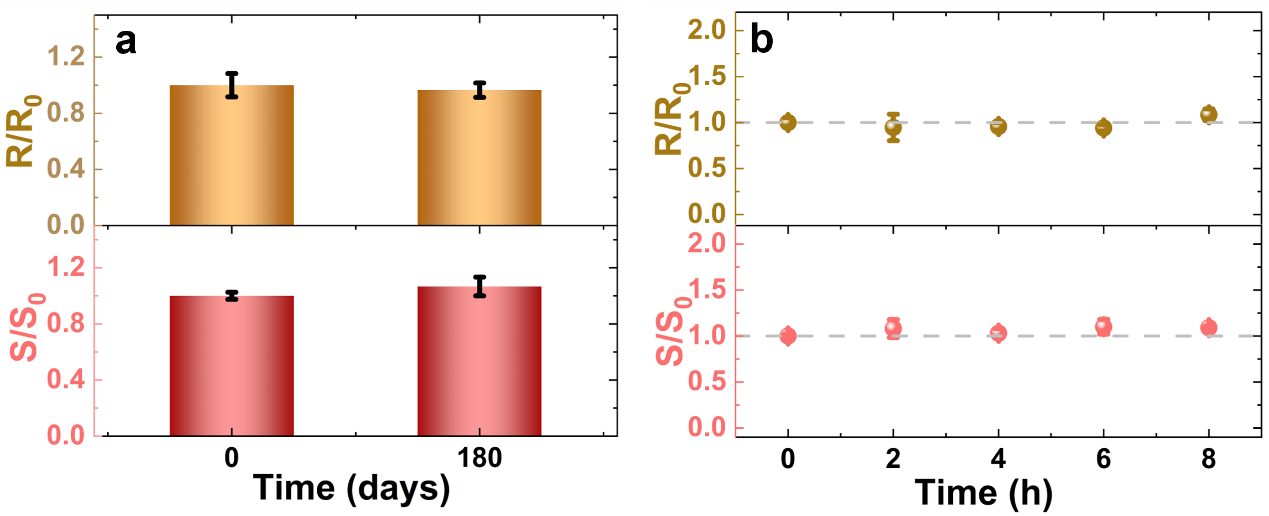


Figure S18. The σ and S of the CNT_pei-doped_ films as a function of storing time in the air at room temperature (a) and a function of Storage time in a heating stage at 86 °C (b).


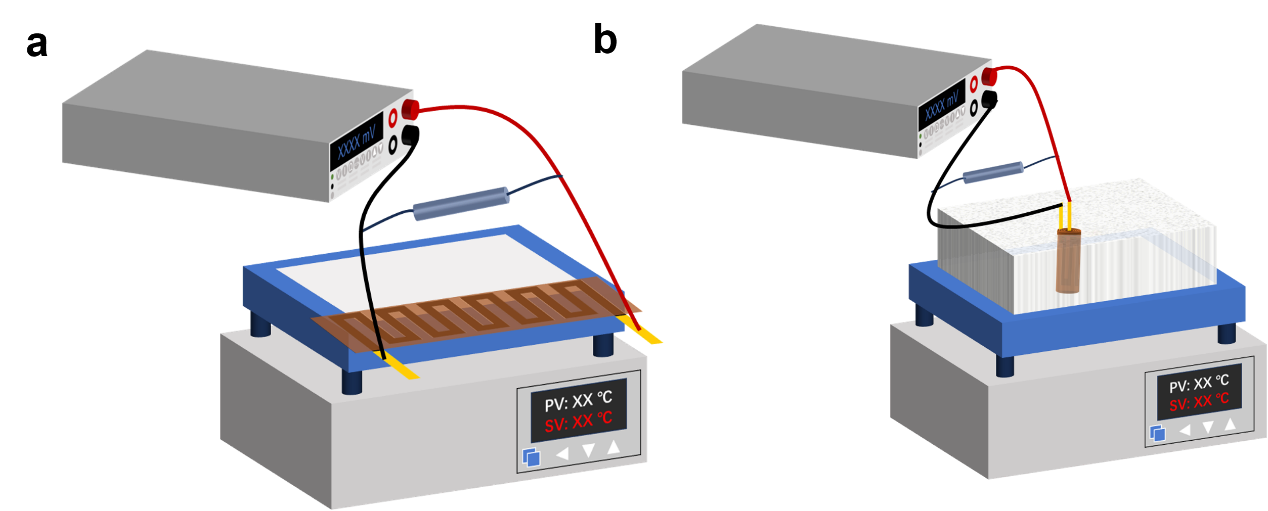


Figure S19. Schematic diagram of the planar (a) and rolled (b) TEG testing.


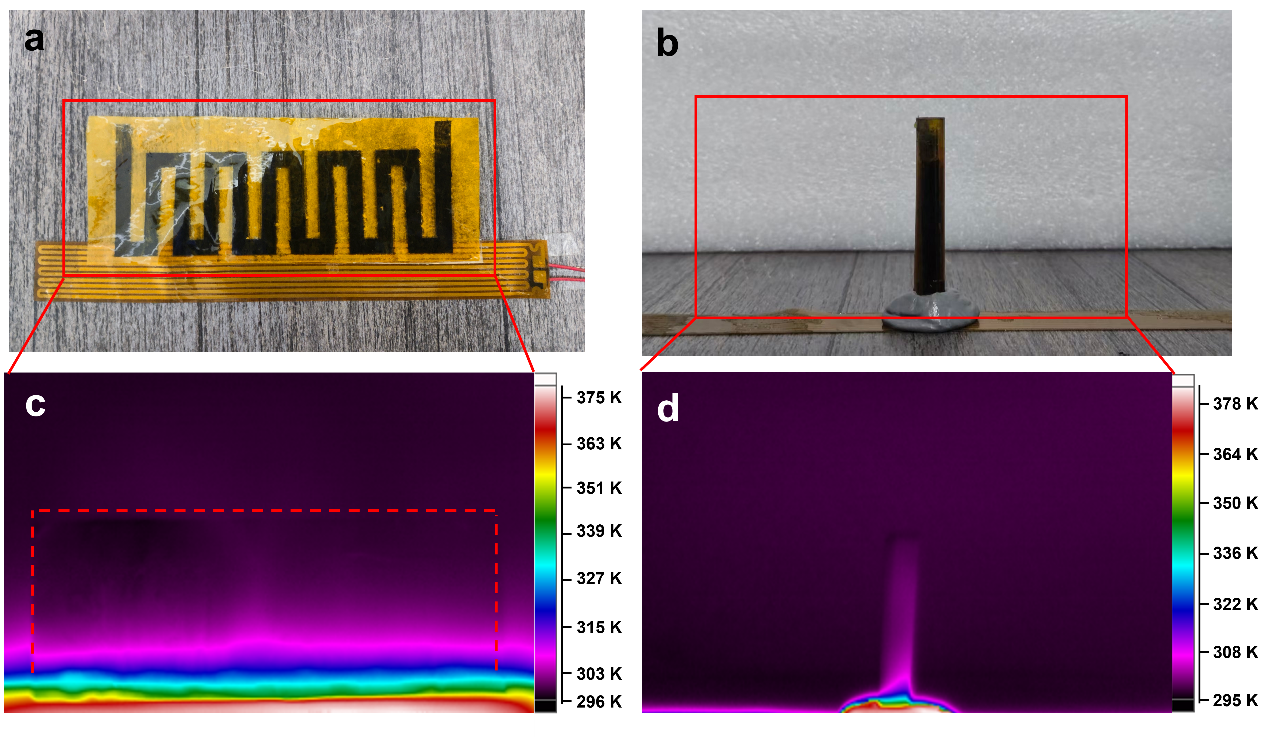


Figure S20. The optical photos of the planar TEG fixed on a heating plate at ~100 ^o^C (a) and the rolled TEG placed in the thermo paste at ~100 ^o^C (b). The infrared images of planar TEG (c) and the infrared images of the rolled TEG (d).


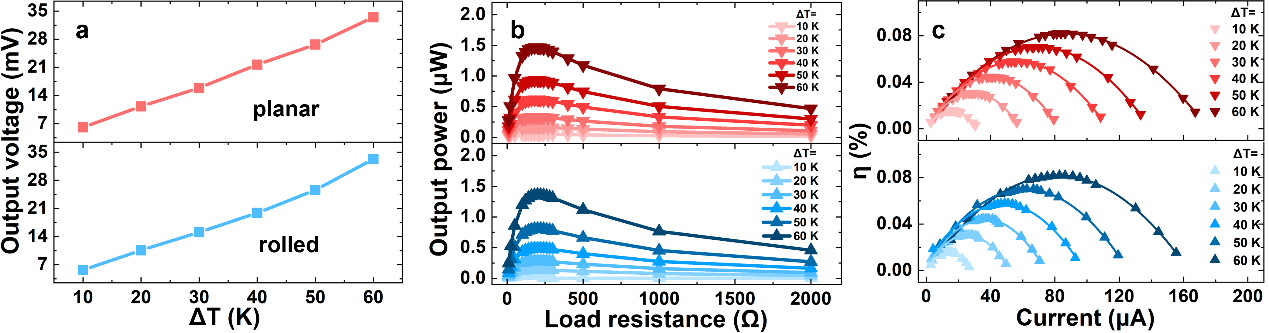


Figure S21. (a) Measured voltage output of the planar and rolled TEG at different temperature differences. (b) The output power of the planar and rolled TEG as a function of load resistance. (c) The conversion efficiency of the planar and rolled TEG at different temperature differences.


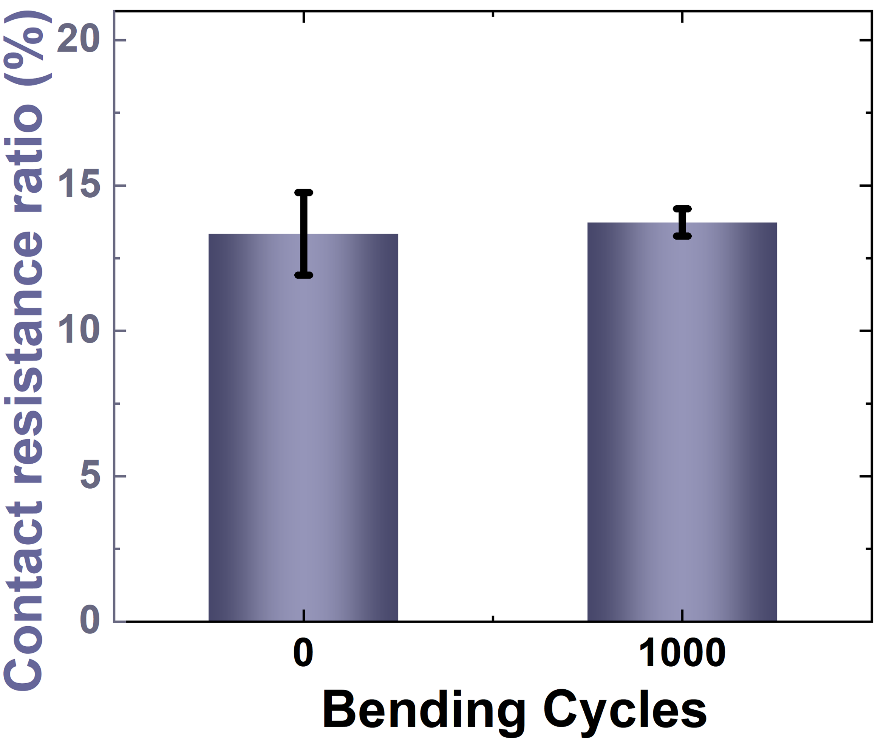


Figure S22. Variations in contact resistance ratio of the single-piece TEG before and after bending in liquid nitrogen. R_cr_ = R_total_ - theoretical R_total_.


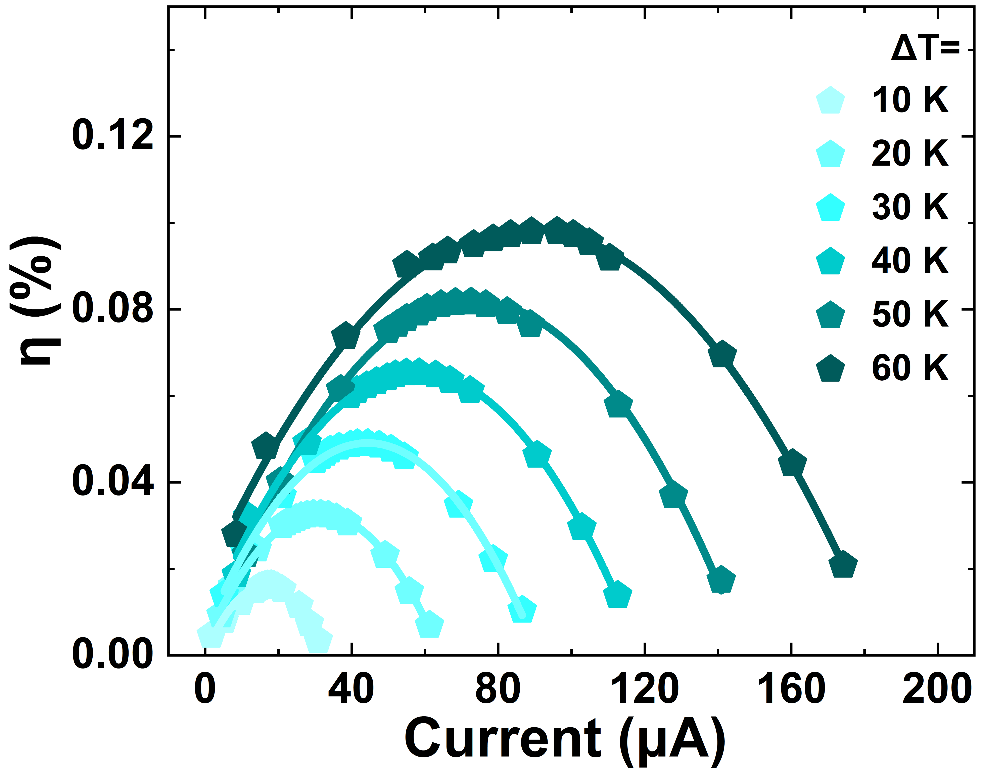


Figure S23. The conversion efficiency of the single-piece TEG at low temperatures.


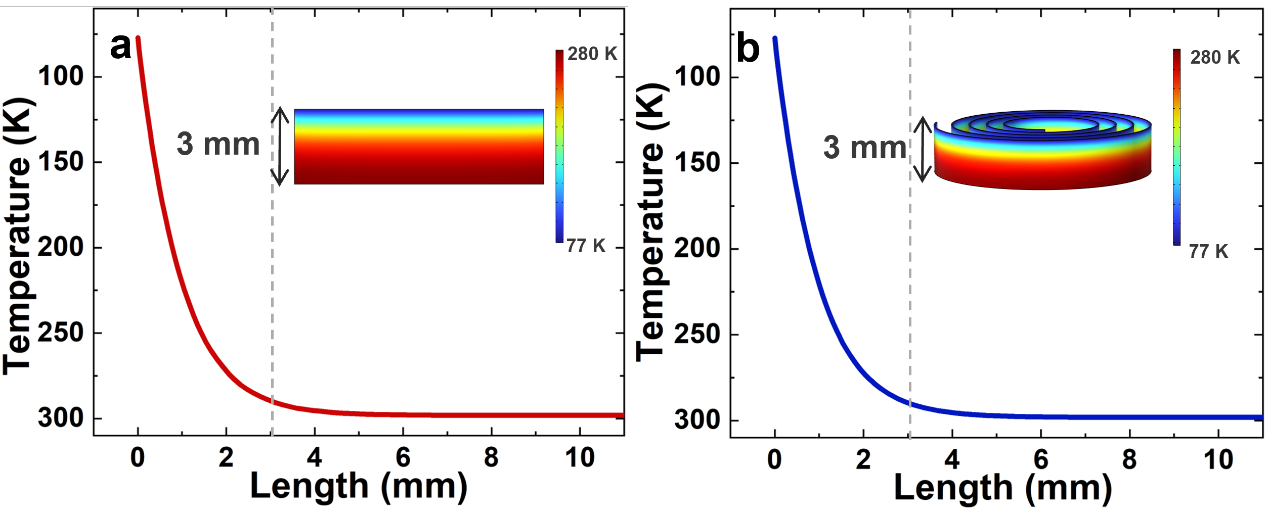


Figure S24. Temperature distribution diagrams and the functional relationships between temperature and distance from the cold end of planar (a) and rolled (b) TEGs.

COMSOL simulations revealed the temperature distribution profiles of the two configurations at a cold-side temperature of 77K. For thermoelectric device (TEG) modules with a length exceeding 3.0 mm, the hot-side temperatures of both planar and rolled configurations approached the ambient temperature (298 K).


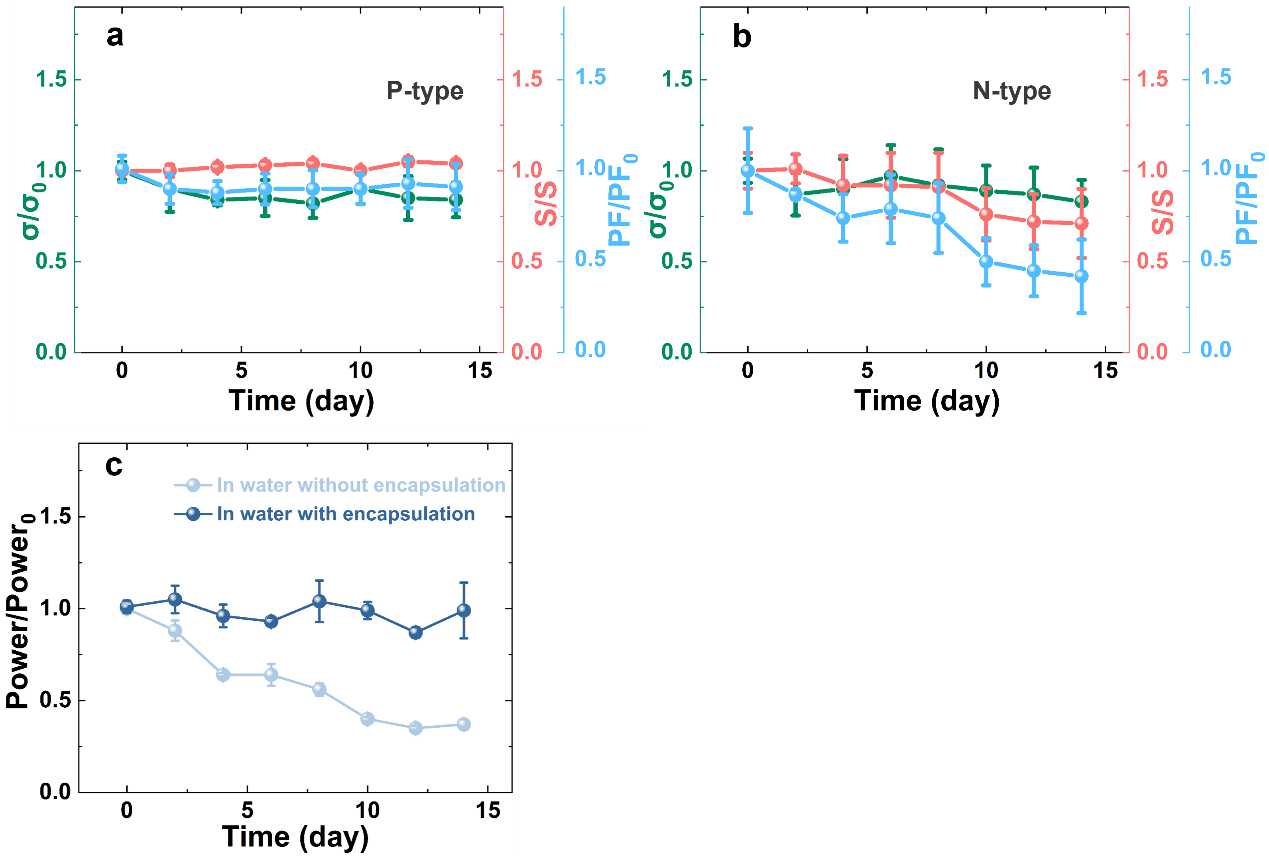


Figure S25. The influence of being immersed in water on the TE properties of (a) the p-type CNT_de-doped/30_ films and (b) the n-type CNT_n-doped_ films. (c) The output power stability of single-piece TEG (in water without encapsulation and in water with encapsulation).


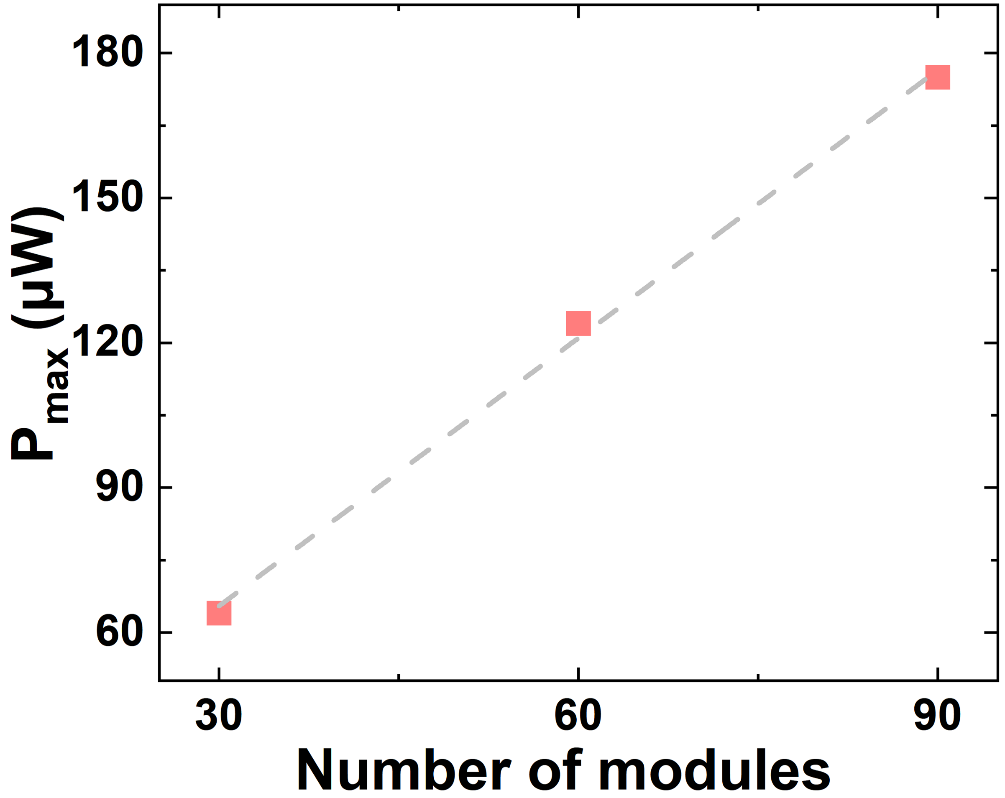


Figure S26. Output power varies with the number of thermoelectric modules.


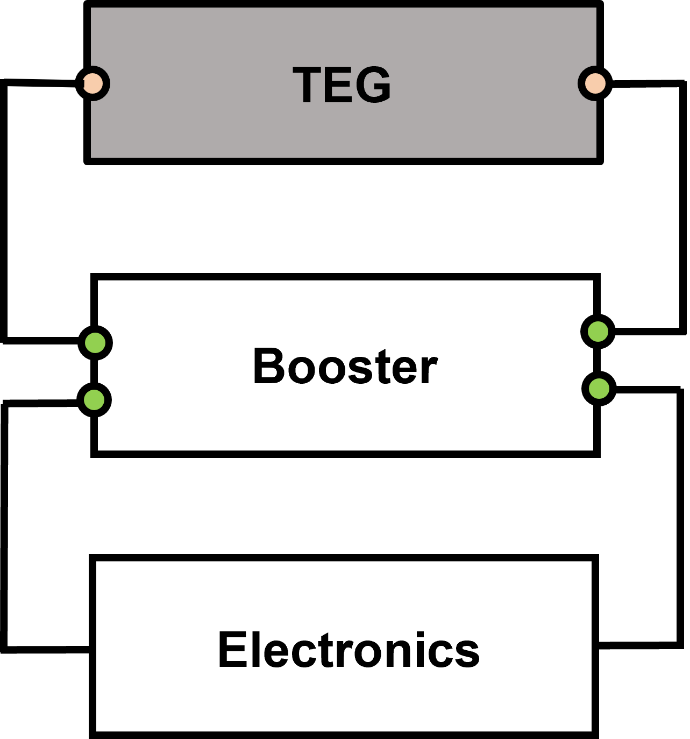


Figure S27. Schematic diagram of a circuit that drives low-power electronic devices through the TEG.


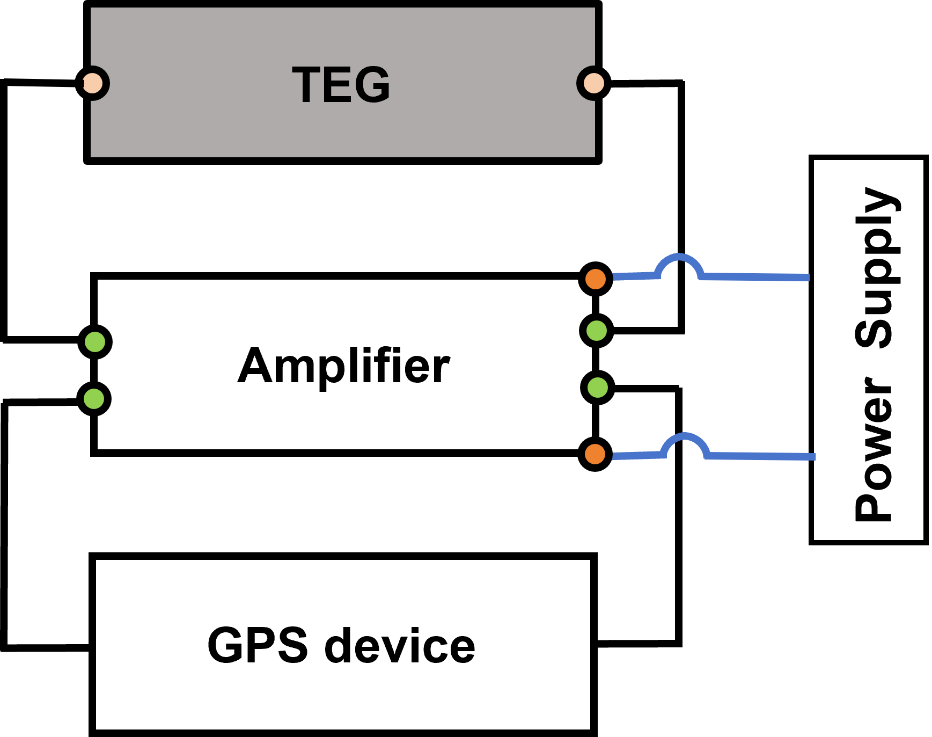


Figure S28. Schematic diagram of a circuit that drives GPS device through the TEG.


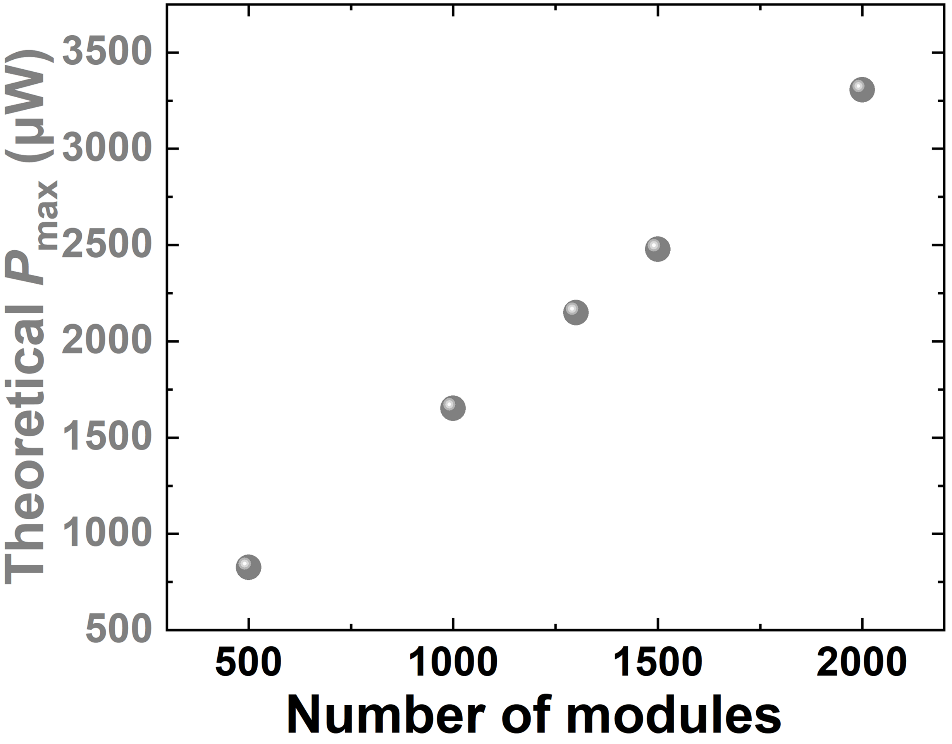


Figure S29. Theoretical maximum output power as a function of the number of modules.

Table S1. Comparison of the TE properties at room temperature of our samples with that of state-of-the-art p-type flexible films in literature.

| Materials | σ(MS·m^-1^) | S ( $\boldsymbol{\mu}$ V·K^-1^) | PF (mW·m^-1^·K^-2^) | Ref. |
| --- | --- | --- | --- | --- |
| CNT_de-doped/30_ film | **~2.8** | **~61** | **~10.5** | **This work** |
| Aligned CNT film | 2.17 | 64 | 9.31 | ^[8]^ |
| MWCNT film | 1.7 | 64 | 7.25 | ^[3a]^ |
| CSA-MWCNT film | ~10 | ~23 | ~4.7 | ^[2]^ |
| few-layer graphene | ~0.07 | ~700 | 4.5 | ^[9]^ |
| CNT composite film | ~0.21 | ~120 | 3.05 | ^[10]^ |
| QL films | 0.19 | 120 | 2.71 | ^[11]^ |
| Willow catkin/CNT | 0.74 | 57 | 2.5 | ^[12]^ |
| SWCNT film | 0.32 | ~290 | 2.48 | ^[13]^ |

Table S2 Circuit simulation data for 15 circuit connections of planar TEG at temperature difference of 60 K.

| **Connection method** | **Voltage (mV)** | **Current (mA)** | **Power(μW)** |
| --- | --- | --- | --- |
| Full parallel | 32.4 | 0.759 | 24.6 |
| Full series | 486.0 | 0.051 | 24.6 |
| S(5p)*3 | 162.0 | 0.152 | 24.6 |
| S(3p)*5 | 97.2 | 0.253 | 24.6 |

**References**

[1] M. C. Biesinger, B. P. Payne, A. P. Grosvenor, L. W. M. Lau, A. R. Gerson, R. S. C. Smart, *Appl. Surf. Sci.* **2011**, *257* (7), 2717.

[2] H. Wang, X. Sun, Y. Wang, K. Li, J. Wang, X. Dai, B. Chen, D. Chong, L. Zhang, J. Yan, *Nat. Commun.* **2023**, *14* (1), 380.

[3] a) K. Li, X. Sun, Y. Wang, J. Wang, X. Dai, Y. Yao, B. Chen, D. Chong, J. Yan, H. Wang, *Small* **2023**, *19* (52), 2304266; b) G. J. Snyder, A. H. Snyder, M. Wood, R. Gurunathan, B. H. Snyder, C. Niu, *Adv. Mater.* **2020**, *32* (25), 2001537.

[4] Y. Dini, J. Faure-Vincent, J. Dijon, *Carbon* **2019**, *144*, 301.

[5] Y. Jung, T. Kim, C. R. Park, *Carbon* **2015**, *88*, 60.

[6] N. Behabtu, C. C. Young, D. E. Tsentalovich, O. Kleinerman, X. Wang, A. W. K. Ma, E. A. Bengio, R. F. ter Waarbeek, J. J. de Jong, R. E. Hoogerwerf, S. B. Fairchild, J. B. Ferguson, B. Maruyama, J. Kono, Y. Talmon, Y. Cohen, M. J. Otto, M. Pasquali, *Science* **2013**, *339* (6116), 182.

[7] A. B. Kaiser, *Rep. Prog. Phys.* **2001**, *64* (1), 1.

[8] H. Wang, K. Li, X. Hao, J. Pan, T. Zhuang, X. Dai, J. Wang, B. Chen, D. Chong, *Nat. Commun.* **2024**, *15* (1), 5617.

[9] N. Xiao, X. Dong, L. Song, D. Liu, Y. Tay, S. Wu, L.-J. Li, Y. Zhao, T. Yu, H. Zhang, W. Huang, H. H. Hng, P. M. Ajayan, Q. Yan, *ACS Nano* **2011**, *5* (4), 2749.

[10] K. T. Park, Y. S. Cho, I. Jeong, D. Jang, H. Cho, Y. Choi, T. Lee, Y. Ko, J. Choi, S. Y. Hong, M.-W. Oh, S. Chung, C. R. Park, H. Kim, *Adv. Energy Mater.* **2022**, *12* (25), 2200256.

[11] C. Cho, K. L. Wallace, P. Tzeng, J.-H. Hsu, C. Yu, J. C. Grunlan, *Adv. Energy Mater.* **2016**, *6* (7), 1502168.

[12] Y. Wang, K. Li, J. Wang, X. Dai, X. Sun, D. Chong, J. Yan, L. Zhang, H. Wang, *J. Mater. Chem. A* **2022**, *10* (48), 25740.

[13] W. Zhou, Q. Fan, Q. Zhang, K. Li, L. Cai, X. Gu, F. Yang, N. Zhang, Z. Xiao, H. Chen, S. Xiao, Y. Wang, H. Liu, W. Zhou, S. Xie, *Small* **2016**, *12* (25), 3407.
